# Agronomic, Nutritional Traits, and Alkaloids of *Lupinus albus*, *Lupinus angustifolius* and *Lupinus luteus* Genotypes: Effect of Sowing Dates and Locations

**DOI:** 10.1021/acsagscitech.3c00581

**Published:** 2024-04-02

**Authors:** Inês M. Valente, André Monteiro, Carla Sousa, Carla Miranda, Margarida R. G. Maia, Carlos Castro, Ana R. J. Cabrita, Henrique Trindade, António J. M. Fonseca

**Affiliations:** † REQUIMTE, LAQV, ICBAS, School of Medicine and Biomedical Sciences, University of Porto, Rua Jorge Viterbo Ferreira, 228, 4050-313 Porto, Portugal; ‡ REQUIMTE, LAQV, Departament of Chemistry and Biochemistry, Faculty of Sciences, University of Porto, Rua do Campo Alegre 687, 4169-007 Porto, Portugal; § Centre for the Research and Technology of Agro-Environmental and Biological Sciences (CITAB), University of Trás-os-Montes and Alto Douro, Quinta de Prados, 5000-801 Vila Real, Portugal

**Keywords:** abiotic stress, alkaloids, biotic stress, lupins, protein, seeds

## Abstract

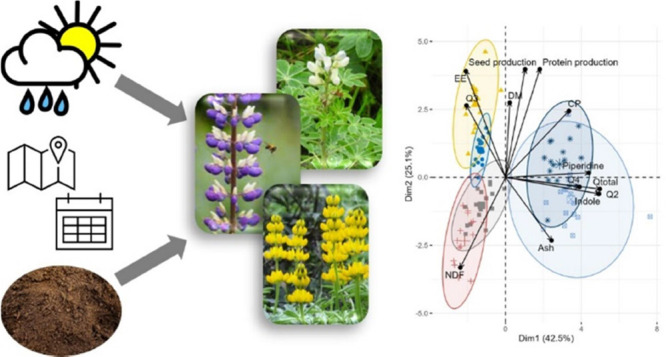

Lupins (*Lupinus* spp.) are legumes with high relevance for the sustainability of agricultural systems as they improve the soil quality, namely, through the fixation of atmospheric nitrogen, and have good adaptability to different climates and soil conditions. Besides, they possess high nutritive value, especially due to the high protein content of the seeds. Nevertheless, the plants’ productivity and metabolism can be influenced by the genotype, the edaphoclimatic conditions, and the sowing practices. In this work, the effect of edaphoclimatic conditions and sowing dates on the productivity, nutritional factors, and alkaloids of the seeds of *L. albus* cv. Estoril, *L. angustifolius* cv. Tango, and *L. luteus* cv. Cardiga was evaluated. High variability in the seeds and protein productions, nutritional traits, and alkaloid content related to the species was observed, along with a significant effect of the location. *Lupinus albus* cv. Estoril showed a good compromise between productivity and low alkaloid content, being an interesting genotype for food and feed use in the conditions of this trial.

## Introduction

Lupins (*Lupinus* spp.) are plants that have been attracting producers and consumers worldwide due to their reduced cost of production, good adaptability to different climate and soil conditions, and high nutritive value, especially due to the high protein content of seeds. From an agronomic perspective, the cultivation of lupins offers several advantages toward an improvement of the soil quality, namely through the fixation of atmospheric nitrogen (N). The lupins roots in association with soil bacteria (rhizobia) convert atmospheric dinitrogen (N_2_) into ammonia that is assimilated by the plant, improving its growth and productivity.^1^ It is estimated that about 50% of the N used in agriculture is supplied by this N_2_ process fixation^2^ which reinforces the importance of these plants for the sustainability of the agricultural sector.

White lupin (*L. albus* L.), narrow-leafed lupin (*L. angustifolius* L.), and yellow lupin (*L. luteus* L.) are three species with a wide range of adaptation to distinct soil and climate conditions supporting adverse conditions like soil acidity and water stress.^3^ They are grown in low-input cropping systems, which, in addition to their adaptation characteristics, make these species interesting solutions for harsh climate-change conditions, contributing to promoting the sustainability of agricultural systems. However, the growth, productivity, and seed yield of lupins vary with species, climatic conditions, soil characteristics, and sowing practices,^4^ the optimal sowing date being therefore dependent on the location. In the Mediterranean region, large climatic variations occur between years, especially rainfall, and these have been aggravated by climate change. The instability within and between legume species is a recognized agronomic issue,^5^ which can be minimized through the uniformization of sowing practices, namely, sowing dates, toward the increase of the crop yield potential and the reduction of heat and drought stresses.

The high protein content in lupin seeds is considered valuable for their use as food and feed, but their consumption may present risks due to the presence of alkaloids with toxic effects.^6^ Indeed, lupins can accumulate considerable amounts of these nitrogen secondary metabolites, particularly quinolizidines, the most abundant alkaloids in lupin plants, which is why most of the research related to this topic is exclusively focused on this class of compounds. Piperidine alkaloids and indole alkaloids can also occur although they are not as common as quinolizidines.^7,8^ The site of alkaloid synthesis in plants is not yet totally established, but several studies have concluded that it occurs predominantly in the aerial green parts of the plant, and the distribution to other parts of the plant occurs by phloem and xylem translocation, accumulating in plant seeds as they mature.^9,10^

During domestication, lupins with a low alkaloid content were selected, and several recessive low-alkaloid mutations have been discovered and genomic resources are available allowing the further decrease of the alkaloid content.^11^ However, cross-pollination in regions where lupins are spontaneous induces a progressive increase of alkaloid in varieties with low alkaloid levels.^12^ Biotechnology^13^ and genetic manipulation of membrane transporters involved in the translocation of quinolizidine alkaloids from phloem to seeds^9^ have been suggested as instrumental for reducing alkaloids in seeds without compromising the levels in vegetative tissues where they are required for plant defense against a variety of pathogenic microorganisms, aphids, and predators, including insects and mammals, and against competing plants by allelopathy.^14−16^ Alkaloids are also recognized to be persistent organic compounds in the environment which implies that lupin crops with high alkaloid content can be responsible for the contamination of the surrounding environment when used as green manure.^3^

The interactions between the genotype, the biosynthesis and translocation of alkaloids in lupin plants, and edaphoclimatic conditions are complex, although a significant impact of the environment on the accumulation of quinolizidine alkaloids in lupins has been described.^17^ The most relevant environmental factors affecting the alkaloids’ production and accumulation are light, drought, and temperature.^3,18^ The biosynthesis of these alkaloids follows a light-regulated cycle, with increased alkaloid production during the day.^19^ Drought stress in lupins also results in increased production of alkaloids but its impact is highly related to the development stage of the plant,^20^ although no strong correlation between rainfall and quinolizidine content in seeds has been observed.^17^ The ambient temperature has an important effect on alkaloid biosynthesis since a small increase in temperature results in a significant increase in alkaloids’ production.^21,22^ The soil characteristics also have an impact on alkaloid synthesis, in particular lower soil pH, potassium deficiency, and higher phosphorus concentrations lead to increased alkaloid content.^23,24^

Although *Lupinus* species are tolerant to water shortages and high temperatures, in many regions where these species are grown, climate changes have led to sharp reductions in precipitation and the occurrence of heat waves during spring. These situations affect the production of alkaloids and increase the relevance of some cultural practices, namely the precocious date of sowing of crops. This work aimed to evaluate the effects of the sowing date and location on the adaptability of three *Lupinus* species (*L. albus* cv. Estoril, *L. angustifolius* cv. Tango, and *L. luteus* cv. Cardiga) grown in two locations with distinct edaphoclimatic characteristics. The performance and metabolic response to abiotic factors were assessed through the measurement of productivity (seeds and protein production), proximate chemical composition, and alkaloid content of the seeds. A preliminary account of part of the work reported here has already been given.^25^

## Materials and Methods

### Reagents

Gramine (99%), (−)-sparteine (≥98%), and (−)-lupinine (100%) were from Sigma (St. Louis, MO). Angustifoline (>98%) and lupanine (>98%) were from Ambinter (Orleans, France). Other reagents used were at least analytical grade.

### Plant Material and Experimental Design

The experimental trials were conducted between September 2018 and June 2019 simultaneously in two locations in the Northeast of Portugal, one at Mirandela (MI, 41.511896, −7.161595) and the other at Vila Real (VR, 41.284747, −7.738875). The monthly rainfall and temperature data are presented in Figure S1 (Supporting Information). The three studied *Lupinus* species were *L. albus* cv. Estoril (Universally Unique Identifier, UUID: NLI/AGR/PT/LUPIN_ALB/206925^26^), and *L. angustifolius* cv. Tango (UUID: NLI/AGR/PL/LUPIN_ANG/207045^26^), both supplied by Fertiprado, Portugal, and *L. luteus* cv. Cardiga (UUID: NLI/AGR/PT/LUPIN_LUT/207074^26^) supplied by INIAV, Instituto Nacional de Investigação Agrária e Veterinária, Elvas, Portugal. The trial was performed using a randomized block design of plots of 10 m^2^ (2.5 × 4.0 m) with 4 replicates and 3 factors: *Lupinus* species, sowing date (D1, D2, D3, and D4), and location (MI and VR). This resulted in 48 plots at each location. Soil characteristics in both locations are presented in Table S1. Sowing was performed on the same day in both locals for each date with approximately 3 weeks intervals between them, from September to November 2018 (D1 18/09/2018, D2 11/10/2018, D3 08/11/2018, and D4 28/11/2018). After the maturation of the grain and with the plants in the same phenological stage, all the plants were harvested in June 2019, and the seeds were collected, dried at 60 °C for 48 h, and grounded at 1 mm for proximal chemical analysis and alkaloids quantification.

### Proximal Chemical Composition

Ground seed samples were analyzed in duplicate according to AOAC^27^ methods for dry matter (DM; ID 934.01), ash (ID 942.05), ether extract (EE; ID 920.39), and Kjeldahl N (ID 954.01) contents. Crude protein (CP) was calculated as Kjeldahl N × 6.25. Neutral detergent fiber (NDF; without sodium sulfite) was also analyzed and expressed exclusive of residual ash.^28^

### Alkaloids’ Determination

Dried *Lupinus* seeds were extracted according to the procedure described by Valente et al.^29^ Briefly, the samples were first extracted under acidic conditions using 5% (v/v) trichloroacetic acid, and after alkalinization of the supernatant, they were extracted by liquid–liquid extraction with dichloromethane. The extract was evaporated until dryness and dissolved in dichloromethane for gas chromatography–mass spectrometry (GC–MS) analysis.

### Statistical Analysis

The analysis of variance (ANOVA) was used to evaluate the significance of the sources of variation considering the effects of genotypes (species/cultivars), the sowing date and location, and all double and triple interactions and was performed in R software (version 4.3.1). Significance was set for *p* < 0.05 and multiple comparisons of means were carried out using the Tukey test with packages multcomp (version1.4-25) and multcompView (version 0.1-9) in R. In order to characterize the effect of the environment on the productivity indexes (seed and protein production) of *Lupinus* species, the Genotype plus Genotype-vs-Environment interaction (GGE) was performed using the “metan” package (1.18.0) of R and presented as a biplot. The terms “genotype” and “environment” were used to designate the species and the interaction location × sowing date, respectively, for the sake of analogy with the existing literature concerning the genotype by environment interaction (G × E).

The characterization of *Lupinus* genotypes by location in terms of productivity (seed and protein), nutritional, and alkaloids profiles was performed by Principal Components Analysis (PCA) using the packages “nFactors” (version 2.4.1.1), “factoextra” (version 1.0.7.999), and “FactoMineR” (version 2.9).

## Results and Discussion

### Effects on Productivity

**Figure 1:**
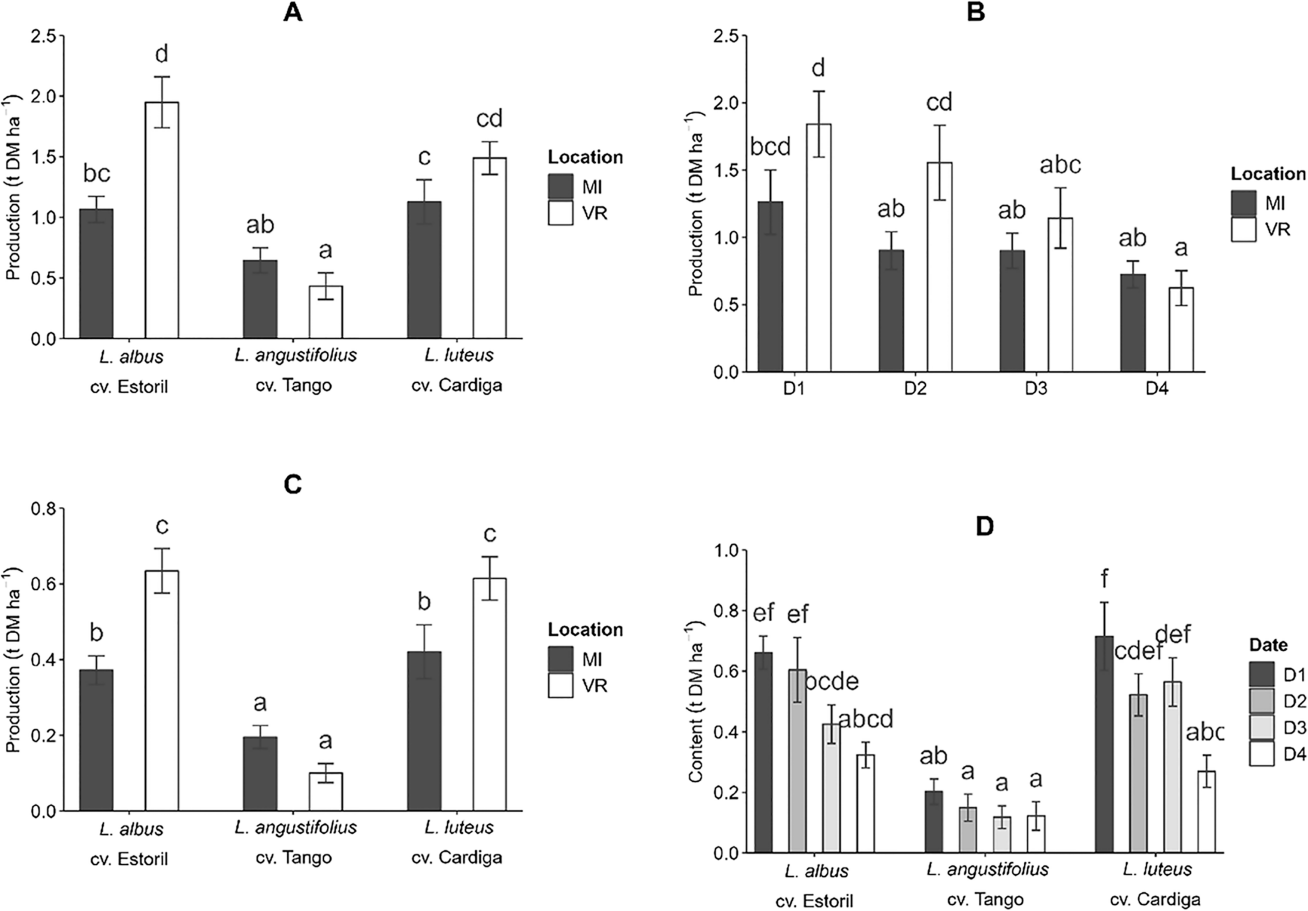
Effects of the interaction genotype × location on seed and protein production (A,C, respectively), sowing date × location on seed production (B), and genotype × sowing date on protein production (D). Different letters in each plot represent significant differences (*p* < 0.05) between values.

The seed yield in the studied *Lupinus* species was affected (*p* < 0.05) by the double interactions genotype × location and sowing date × location, while the protein yield was influenced by the interactions genotype × sowing date and genotype × location (Table S2). For both productivity indexes, it was observed that genotypes were responsible for most of the variation (30.2 and 38.5% for seed and protein production, respectively), followed by the sowing date (17.6 and 14.2% for seed and protein production, respectively). *Lupinus albus* cv. Estoril showed the highest seed productivity at VR (1.95 t DM ha^–1^, Figure 1A) and was the only genotype for which the seed yield was affected by the location. However, in terms of protein production, both *L. albus* cv. Estoril and *L. luteus* cv. Cardiga were affected by the sowing location, VR being more favorable to protein production for these species (Figure 1C). *Lupinus angustifolius* cv. Tango was the species showing the lowest values of seeds and protein production, similar between locations (0.645 and 0.431 t DM ha^–1^ for seeds; 0.195 and 0.100 t DM ha^–1^ for protein). At both locations, *L. albus* cv. Estoril and *L. luteus* cv. Cardiga produced similar seeds and protein productivity (Figure 1A,C).

The seed yield in different *Lupinus* genotypes was affected by the sowing date × location (*p* = 0.021, Table S2), with significantly different values only for D2 for which a higher seed production was observed at VR (Figure 1B). The protein production was higher in the early sowing dates but only for *L. albus* cv. Estoril and *L. luteus* cv. Cardiga (*p* = 0.021, Figure 1D).

**Figure 2:**
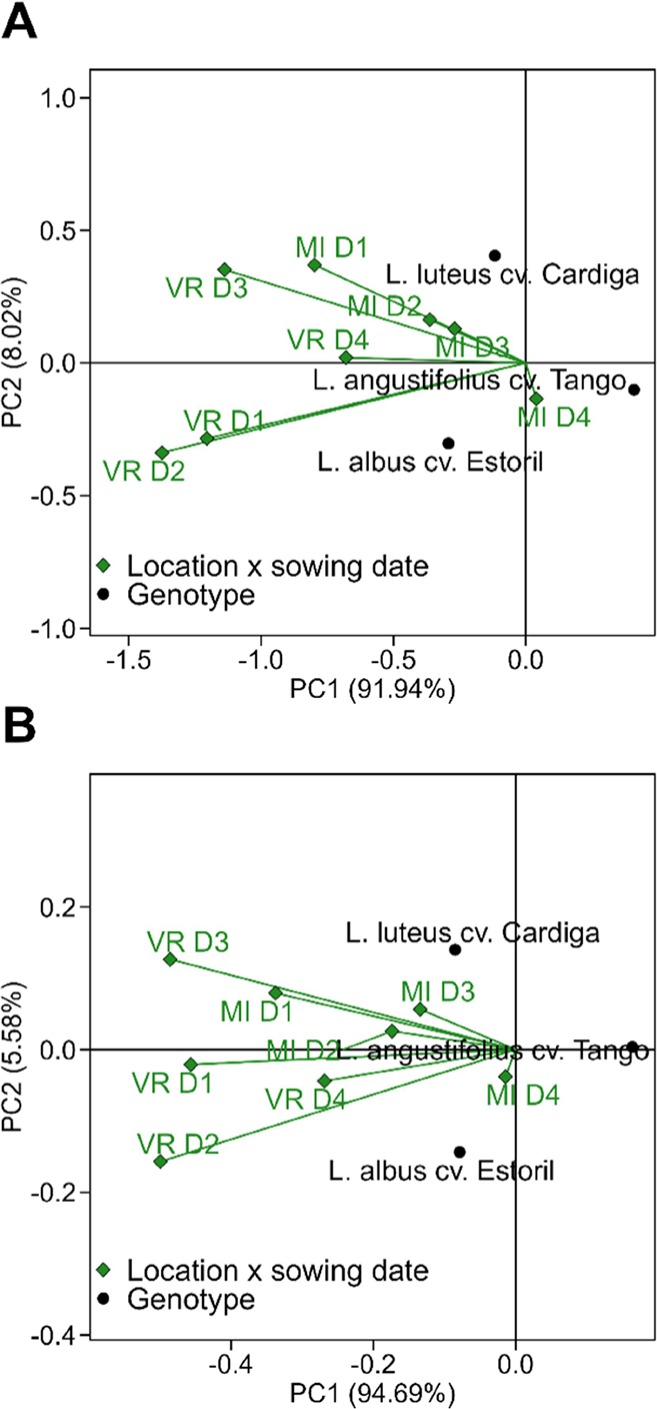
Genotype plus Genotype-vs-Environment interaction (GGE) biplot for (A) seed production and (B) protein production for the studied *Lupinus* genotypes.

The impact of the environment on the productivity indexes of the studied *Lupinus* genotypes is shown in the Genotype plus Genotype-vs-Environment interaction (GGE) biplots in Figure 2. The first component explained 91.9 and 94.7% of the variance for seed and protein production, respectively, and distinguished *L. albus* cv. Estoril and *L. luteus* cv. Cardiga from *L. angustifolius* cv. Tango.

*Lupinus albus* and *L. luteus* genotypes were shown to be susceptible to the edaphoclimatic differences between MI and VR, being the later location more adequate to high seeds and protein yield for these genotypes, and especially in the early sowing dates (Figure 2). The high accumulated rainfall and lowest maximum temperatures during flowering and pod and seed growth at VR (Figure S1) could explain the increased grain productivity at VR of *L. albus* cv. Estoril and *L. luteus* cv. Cardiga and suggest the poor adaptation of *L. angustifolius* to the edaphoclimatic conditions of both locations in this agronomic year. Although some genetic variations for drought tolerance can occur,^30^ water deficit can impair lupin production, especially during flowering and pod growth.^31^

Annicchiarico and Iannucci^32^ observed that the seed yield of *L. albus* genotypes was differently affected by early or late autumn sowing in two different locations in Italy related to climate differences. The effect of delaying the sowing date from autumn to winter in the United Kingdom has been shown to result in a decrease in the productive yields of *L. albus* caused by the effect of the weather conditions in plant development.^33^ In fact, *L. albus* narrow optimal sowing time is well recognized.^34^ The differences in temperature and rainfall between sowing dates could be the cause for the results obtained in the present work, especially due to the high rainfall observed in November (Figure S1), which corresponds to the sowing dates D3 and D4. In fact, the effect of water logging on the growth and infection susceptibility of *L. albus* is reported.^31^ Besides, later sowing dates led to later flowering, the most susceptible genotypes being affected by the decreasing rainfall and increasing temperatures verified in May and June when compared to March and April (Figure S1).

The absence of the sowing date and location effects on *L. angustifolius* cv. Tango productions indicates that the edaphoclimatic conditions of this trial did not affect the seed and protein yield in this genotype. These results suggest a good adaptability of this genotype to the conditions of the trial and a good stability across locations.

### Effects on Nutritional Composition of Seeds

The proximate chemical composition (DM, ash, EE, NDF, and CP) of the seeds showed to be only affected (*p* < 0.05, Table S3) by the interaction species × sowing location, indicating the highest importance of the edaphoclimatic conditions at the expense of the sowing date on the chemical composition of *Lupinus* seeds. The seeds’ DM showed to be different (*p* < 0.05) for *L. angustifolius* in which a slightly higher DM was determined in seeds of plants grown at MI (91.1%, Figure S2A). The effect of sowing date was also significant (*p* < 0.05) for DM; late sowing dates D3 and D4 were different between each other (Figure S2B).

**Figure 3:**
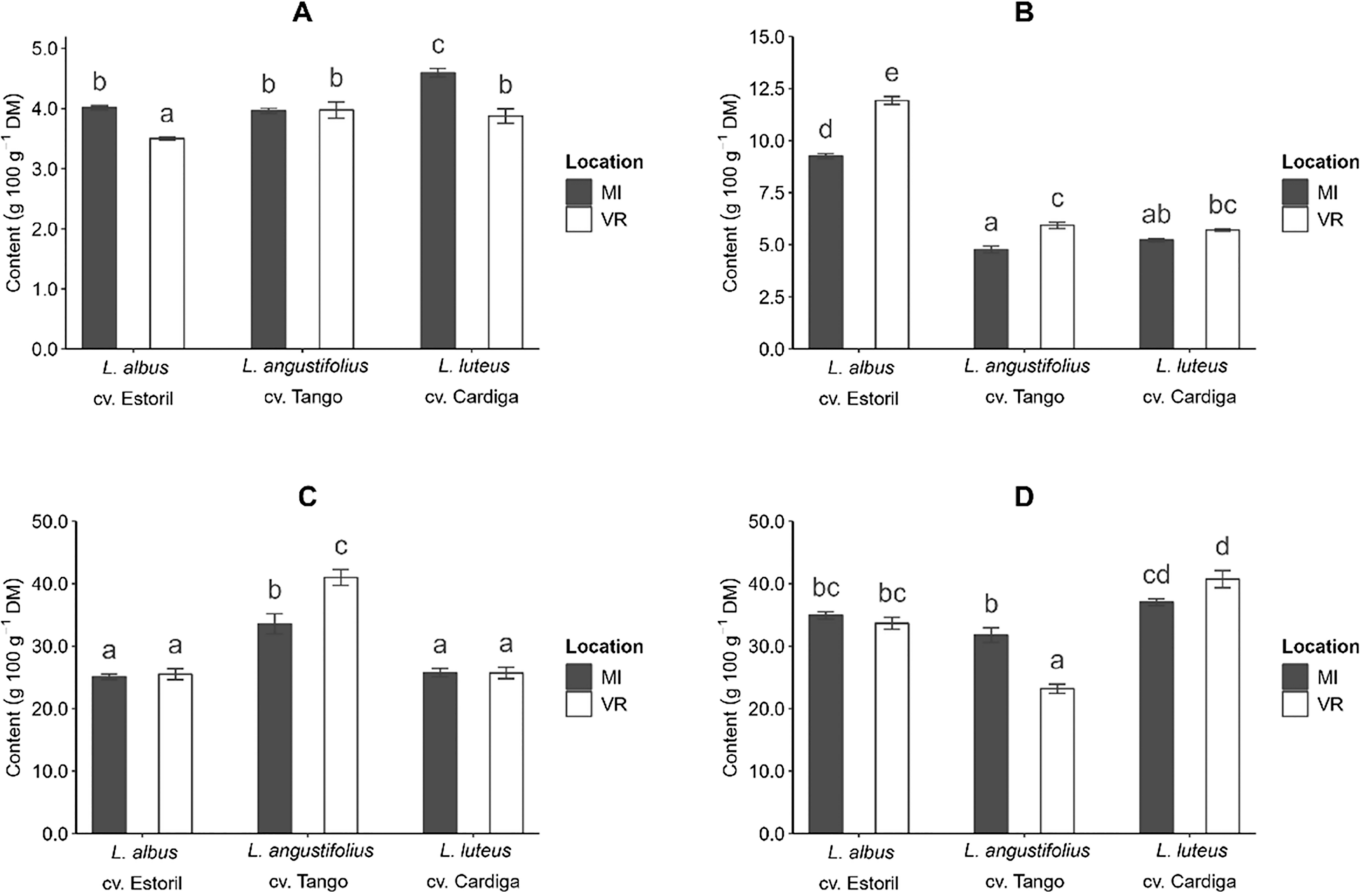
Effect of the interaction genotype × location on the *Lupinus* seeds’ (A) ash (g 100 g^–1^ DM), (B) ether extract (EE, g 100 g^–1^ DM), (C) neutral detergent fiber (NDF, g 100 g^–1^ DM), and (D) crude protein (CP, g 100 g^–1^ DM). Different letters in each plot represent significant differences (*p* < 0.05) between values.

The species was the main source of variation of the results for EE, NDF, and CP (between 51.4 and 85.6%), while for ash content, the contribution of species and location was very similar (18.3 and 20.0%, respectively). The ash content of seeds showed values between 3.50 and 4.6 g 100 g^–1^ DM and sowing at MI resulted in higher ash content for *L. albus* and *L. luteus* genotypes; *L. angustifolius* was not affected (*p* > 0.05) by the sowing location (Figure 3A). At MI higher mineral content of the soil was verified (Table S1) which can explain the highest ash content in the lupin seeds at that location. The fact that only *L. albus* and *L. luteus* showed differences between locations suggests a dependency on genetic factors, yet information in the literature supports this observation.

The EE of *L. albus* cv. Estoril was the highest of the three studied genotypes (9.26 and 11.9 g 100 g^–1^ DM, Figure 3B). The effect of the location on the EE content was observed for *L. albus* cv. Estoril and *L. angustifolius* cv. Tango, for which the highest values were verified at VR. The NDF and CP contents were only different between locations for *L. angustifolius* cv. Tango, being NDF higher at VR (40.5 g 100 g^–1^ DM, Figure 3C) and CP higher at MI (31.8 g 100 g^–1^ DM, Figure 3D). Still, higher CP contents were obtained for *L. luteus* cv. Cardiga (37.1 and 40.7 g 100 g^–1^ DM), in accordance with the results previously reported that showed the highest protein content compared to some *L. albus* and *L. angustifolius* genotypes.^29^ The sowing date was also responsible for small variations in the NDF content of *L. angustifolius* cv. Tango, with date D4 having the highest value (41.7 g 100 g^–1^ DM, Figure S3).

Calabrò et al.^35^ pointed out the influence of rainfall and temperature on the nutritional composition of *L. albus* seeds, but due to a high effect of the genotype on the results of the present work, the effect of the edaphoclimatic conditions on the nutritional traits of lupin seeds cannot be totally understood.

### Alkaloids’ Identification in *Lupinus* Seeds

A total of 27 alkaloids were identified in the studied *Lupinus* genotypes distributed by three main chemical classes: indoles, piperidines, and quinolizidines (bicyclic, tricyclic, and tetracyclic). The list of the identified alkaloids and the corresponding information on retention indexes (RI), retention time (Rt), and *m*/*z* values are presented in Table 1, and the chemical structures in Figure S4.

**Table 1:** List of Alkaloids Identified in *Lupinus* Species Grouped by Chemical Class and GC–MS Details^a^

alkaloid	RI	Rt/min	M^−^	*m*/*z* (relative intensity)	detected in
Indoles					
gramine (1)	1622	7.4	174	130 (100), 174 (30), 131 (21), 77 (10), 103 (7)	*L. luteus* cv. Cardiga
gramine derivative		13.7	214	130 (100)	*L. luteus* cv. Cardiga*
Piperidines					
smipine (2)	1580	7.1	180	109 (100), 96 (74), 151 (14), 112 (11), 163 (2)	*L. albus* cv. Estoril*
*N*-methylammodendrine (3)	1835	10.9	222	98 (100), 222 (72), 150 (57), 207 (12), 136 (47)	*L. luteus* cv. Cardiga
ammodendrine (4)	1865	11.6	208	165 (100), 136 (60), 123 (60), 208 (55), 191 (50)	*L. albus* cv. Estoril
					*L. luteus* cv. Cardiga
Bicyclic Quinolizidines					
lupinine (5)	1420	4.6	169	152 (81), 169 (84), 138 (76), 97 (70), 83 (100)	*L. luteus* cv. Cardiga
lusitanine (6)	1875	12	208	136 (100), 166 (88), 110 (72), 208 (45), 179 (30)	*L. luteus* cv. Cardiga
feruloyllupinine (7)		42.6	345	152 (100), 168 (40), 345 (20), 136 (6)	*L. luteus* cv. Cardiga*
Tricyclic Quinolizidines					
albine (8)	1900	12.4	232	191 (100), 110 (50), 149 (40), 122 (35), 232 (20)	*L. albus* cv. Estoril
*iso*-angustifoline (9)	2033	14.9	234	193 (100), 112 (70), 150 (10), 55 (30), 94 (20)	*L. albus* cv. Estoril*
					*L. angustifolius* cv. Tango*
tetrahydrorhombifoline (10)	2050	15.1	248	207 (100), 58 (80), 112 (15), 108 (10), 248 (1)	*L. albus* cv. Estoril*
					*L. angustifolius* cv. Tango*
angustifoline (9)	2083	15.8	234	193 (100), 112 (85), 150 (15), 55 (20), 94 (11)	*L. albus* cv. Estoril*
					*L. angustifolius* cv. Tango
11,12-seco-12,13-didehydromultiflorine (11)	2215	18.2	246	205 (50), 58 (100), 110 (15), 94 (20), 246 (10)	*L. albus* cv. Estoril
Tetracyclic Quinolizidines					
α-iso-sparteine (12)	1710	8.6	234	98 (100), 137 (57), 193 (22), 234 (40), 150 (15)	*L. luteus* cv. Cardiga*
					*L. albus* cv. Estoril*
sparteine (12)	1785	9.8	234	137 (100), 98 (90), 234 (44), 193 (25), 84 (10)	*L. angustifolius* cv. Tango*
					*L. luteus* cv. Cardiga
β-iso-sparteine (12)	1830	10.7	234	98 (62), 137 (100), 193 (16), 234 (20), 150 (13)	*L. luteus* cv. Cardiga*
7-hydroxy-β -isosparteine (13)	1966	13.9	250	98 (100), 166 (31), 84 (30), 153 (26), 250 (21)	*L. luteus* cv. Cardiga*
dihydromultiflorine (14)	2100	15.9	248	134 (100), 136 (80), 150 (20), 248 (15), 219 (15)	*L. albus* cv. Estoril*
α -isolupanine (15)	2105	16.2	248	136 (100), 248 (50), 149 (50), 98 (30), 219 (5)	*L. albus* cv. Estoril
					*L. angustifolius* cv. Tango
lupanine (15)	2165	17.1	248	136(100), 149(60), 248(40), 150(34), 219(8)	*L. albus* cv. Estoril
					*L. angustifolius* cv. Tango
7-hydroxylupanine (16)	2275	19.1	264	98 (100) 152 (80) 84 (51) 264 (42) 150 (33)	*L. luteus* cv. Cardiga*
multiflorine (17)	2310	20.1	246	134 (100), 246 (65), 148 (20), 110 (15), 217 (5)	*L. albus* cv. Estoril
17-oxolupanine (18)	2350	21.1	262	150 (100), 262 (40), 110 (30), 234 (10), 55 (20)	*L. angustifolius* cv. Tango*
13α -hydroxylupanine (19)	2400	22.3	264	152 (100), 165 (40), 264 (40), 246 (40), 134 (30)	*L. albus* cv. Estoril
					*L. angustifolius* cv. Tango
13α -angelolyoxylupanine (20)	2733	34.6	346	246 (100), 134 (30), 148 (15), 112 (12), 55 (10)	*L. albus* cv. Estoril
13α-tigloyloxylupanine (21)	2753	36.4	346	246 (100), 134 (30), 148 (15), 112 (12), 55 (10)	*L. albus* cv. Estoril*
13-tigloyloxymultiflorine (22)	2955	39.4	344	244 (56), 344 (13), 132 (100), 149 (40)	*L. albus* cv. Estoril*

a RI – Retention indices described in the literature.^8^ Rt – Experimental retention time. Gramine, lupinine, sparteine, angustifoline, and lupanine were identified by comparison with authentic standards, the other compounds were tentatively identified and NIST 05 Library Database.^56^ * denotes below the limit of quantification.

Each lupin species has a characteristic alkaloid profile, as will be discussed further in the text. The structural diversity of alkaloids produced by lupins should be considered as different pharmacological/toxic effects may be expected especially relating to the indole/piperidine/quinolizidine nucleus, with an impact on its use as food and feed. This information is also important for selecting the varieties that are better adapted to biotic and abiotic stresses. The alkaloid content and diversity are highly variable among *Lupinus* species and cultivars.^36,37^

Indole alkaloids are synthesized from *L*-tryptophan, while the remaining alkaloids detected in *Lupinus* are derived from the amino acid *L*-lysine.^18^ In the literature, only the major alkaloids (quinolizidines) are usually reported and are often described as “lupin alkaloids”, the most relevant ones in *Lupinus* due to their high levels in this genus.^18^ This class occurs mostly in the Leguminosae family and is responsible for the protection of plants against insect pests.^38^ However, the high levels of quinolizidine alkaloids in *Lupinus* seeds are a high concern for human and animal consumption due to their bitter taste and high toxicity.^6^ The biosynthesis of quinolizidine alkaloids occurs via decarboxylation of *L*-lysine, forming the major structural compounds lupinine (bicyclic), sparteine, lupanine, and multiflorine (tetracyclic). These alkaloids can be further modified by dehydrogenation, oxygenation, hydroxylation, glycosylation, or esterification forming a wide variety of related quinolizidines,^18^ as those identified in the present work (Figure S4).

### Alkaloids Profile Differences between Genotypes

In this study, the main effects of *Lupinus* genotypes, sowing date, and sowing location on the alkaloids’ concentration were evaluated considering the main alkaloid classes (indole, piperidine, and quinolizidine), the individual alkaloids, and the total content of alkaloids in the seeds. Due to the relevance of quinolizidine alkaloids in the *Lupinus* genus and the different biological activities among compounds, the occurrence of this class of alkaloids in the seeds was also evaluated considering their chemical structure, according to the grouping presented in Figure S4. The ANOVA results for the alkaloids content by a group of alkaloids are presented as Supporting Information (Tables S5–S8).

**Figure 4:**
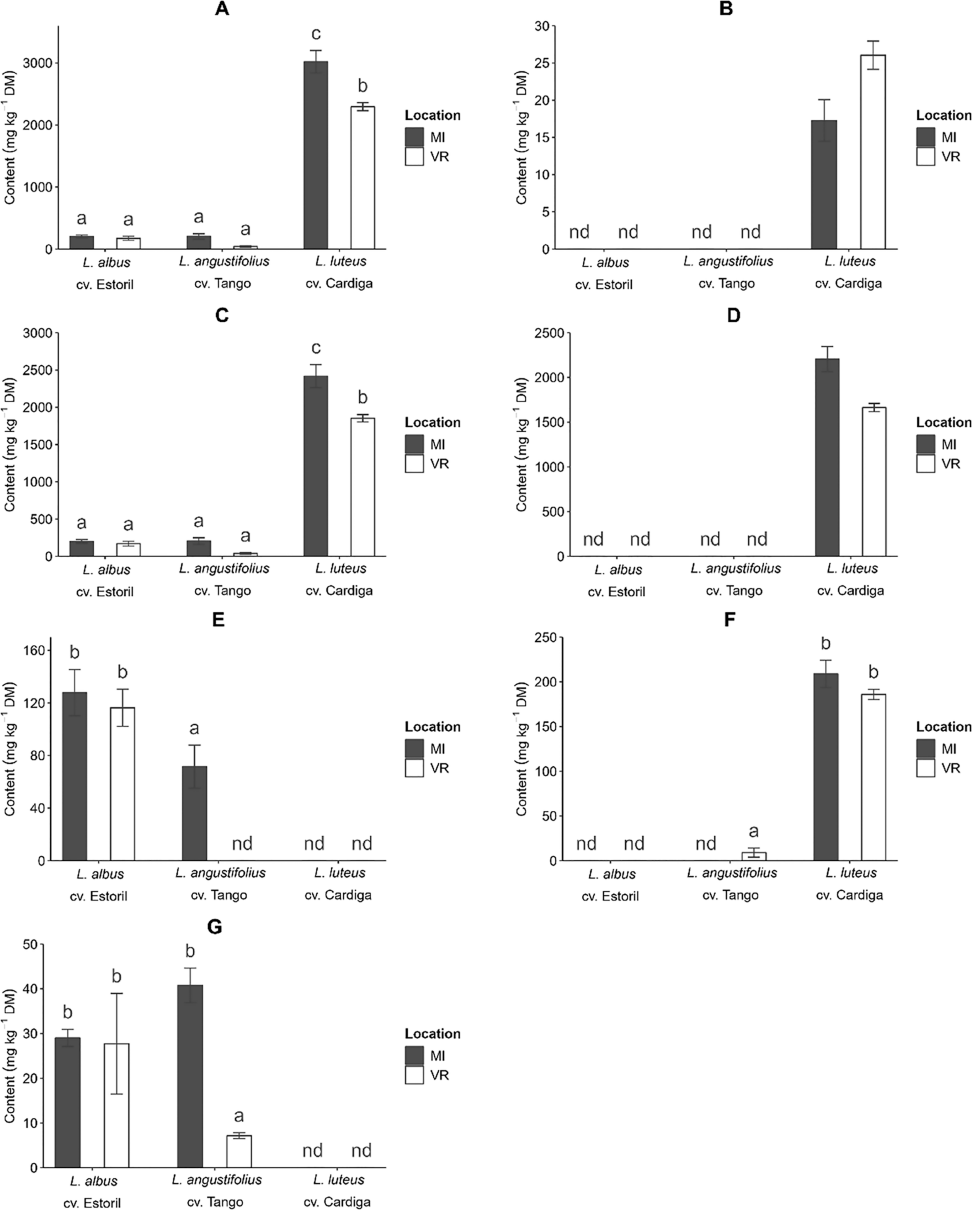
Effect of the interaction genotype × location on (A) total alkaloids, (B) piperidine alkaloids, (C) total quinolizidine alkaloids, (D) bicyclic quinolizidine alkaloids, (E) tricyclic quinolizidine alkaloids, (F) sparteine, and (G) lupanine content (mg kg^–1^ DM) of the *Lupinus* seed. Bars of the same factor not sharing the same letter differ significantly (*p* < 0.05).

The total content of alkaloids in *Lupinus* seeds was significantly (*p* < 0.05) affected by the interaction species × sowing location (Table S8), with *L. luteus* cv. Cardiga sown at MI presented the highest value (3021 mg kg^–1^ DM), followed by that sown at VR (2296 mg kg^–1^ DM); *L. albus* cv. Estoril and *L. angustifolius* cv. Tango showing similar values (40.7–210 mg kg^–1^ DM; Figure 4A). *Lupinus luteus* cv. Cardiga is a traditional Portuguese cultivar with known high alkaloid content and susceptible to environmental conditions.^29^ The increased production of alkaloids in *Lupinus* plants caused by the sowing location has been related to various abiotic stress conditions, such as higher daily mean temperatures,^21^ and drought stress.^20^ Differences in the mean temperatures and rainfall in both locations were observed (Figure S1) showing that at MI slightly higher temperatures and considerably lower rainfall were registered compared to those at VR, which can explain the highest accumulation of alkaloids in the seeds of *L. luteus* cv. Cardiga in MI.

The alkaloid profiles between species were shown to be very different (Table 1). *Lupinus luteus* cv. Cardiga was the only species with indole and bicyclic quinolizidine alkaloids. Piperidine alkaloids were quantified in *L. albus* cv. Estoril and *L. luteus* cv. Cardiga, while bicyclic quinolizidine alkaloids only occurred in *L. albus* cv. Estoril and *L. angustifolius* cv. Tango. Tetracyclic quinolizidines were present in all three studied genotypes. The complete data set showing the individual alkaloids content by genotype, sowing date, and location are presented in Table S4.

In *L. luteus* cv. Cardiga, the most prominent classes accounting for the high levels of total alkaloids are quinolizidines (bicyclic 1517–2506 mg kg^–1^ DM, and tetracyclic 172–249 mg kg^–1^ DM), and indoles (370–681 mg kg^–1^ DM). In fact, quinolizidines are the most abundant types of alkaloids in the seeds, composing more than 80% of the total alkaloid content in all the studied species. The higher levels of quinolizidine alkaloids observed in *Lupinus* when compared to the remaining chemical classes confirm their high relevance for the assessment of the antinutritional value of the seeds. Therefore, most of the reports refer to the total alkaloid content as the concentration of quinolizidines.^36^ In the next sections, a detailed description and discussion on the occurrence of alkaloids accounting for the total alkaloids content by chemical class and their dependence on the sowing date and sowing location will be done.

### Indole Alkaloids

*Lupinus luteus* cv. Cardiga was the only genotype that presented indole alkaloids (Table 1). Indole alkaloids are derived from *L*-tryptophan^39^ and are not common in most *Lupinus* plants; only a few studies reported the presence of indole alkaloids in this genus.^15,40−42^ The identified alkaloids of this group were gramine and a gramine derivative (Table 1). The gramine derivative was first described by Adhikari et al.^15^ in the leaves of the varieties *L. luteus* cv. Wodjil and Teo. In the present work, only gramine was quantified. The interaction genotype × sowing date × location significantly (*p* < 0.05, Table S5) affected the gramine content (i.e., the indole alkaloids content) in the seeds. For the variation, date and location showed a minor contribution (0.239 and 1.27%, respectively).

**Figure 5:**
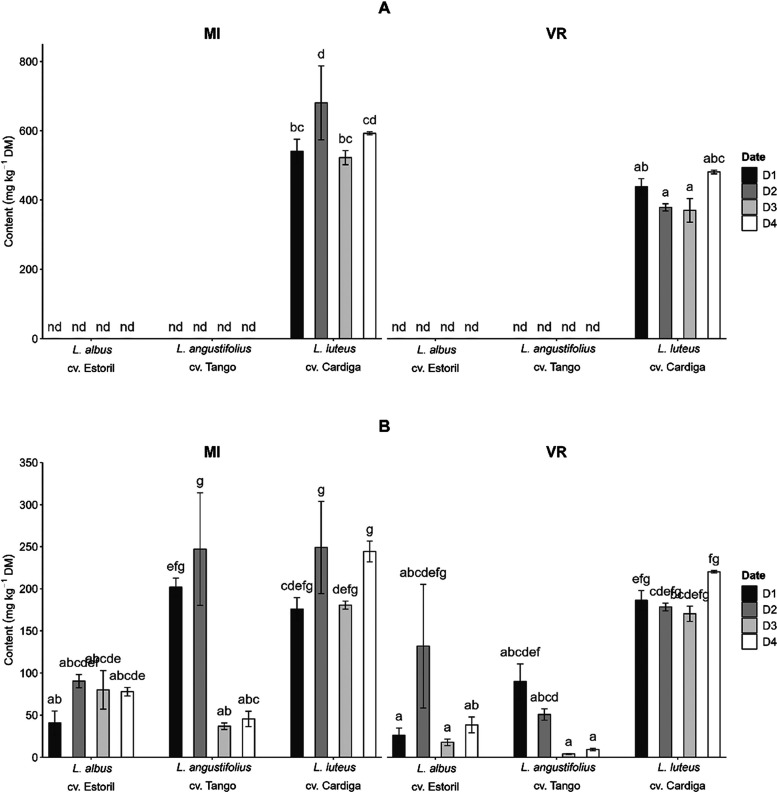
Effect of the interaction genotype × sowing date × location on (A) gramine and (B) tetracyclic quinolizidine alkaloids content (mg kg^–1^ DM) of *Lupinus* seed. Bars of the same factor not sharing the same letter differ significantly (*p* < 0.05).

Gramine varied between 370 and 681 mg kg^–1^ DM (Figure 5A), corresponding to 17.5–21.0% of the total alkaloids content, with the highest value being attributed to sowing at MI on D2. Although at VR, the results were not different (*p* > 0.05) between sowing dates, variations were verified at MI, yet without any noticeable correlation with a specific soil or climate variable.

The occurrence of gramine in *Lupinus* is highly variable with the species and genotypes. Wink, Meißner, and Witte^8^ determined gramine in concentrations above 1% of the total alkaloid content in a few European and North American *Lupinus* species, but not in *L. albus*, *L. angustifolius* nor *L. luteus*. However, other authors reported the presence of gramine in *L. luteus* seeds at levels that comprise those determined in the present work (7–6507 mg kg^–1^).^15,40,42^

Gramine is known to have a high toxic effect on aphids which can potentially confer a good plant tolerance to these insects.^16^ A concentration of gramine above 15 μg g^–1^ was shown to be correlated with a high resistance to aphids^15^ which suggests that *L. luteus* cv. Cardiga can be highly protected from these insects due to the high levels of gramine with good adaptability of the plant to biotic stress.

### Piperidine Alkaloids

Piperidine alkaloids *N*-methylammodendrine and ammodendrine were quantified in *L. luteus* cv. Cardiga. These alkaloids are toxic to adult mammals and produce deformities in neonatal individuals.^43^*N*-methylammodendrine was affected (*p* < 0.05, Table S6) by the interaction genotype × sowing date × location, while ammodendrine was affected by genotype × date and genotype × location.

The methylated derivative of ammodendrine, *N*-methylammodendrine, was quantified only in *L. luteus* cv. Cardiga (3.33–3.81 mg kg^–1^ DM) in sowing date D2 at MI, and in D1, D2, and D4 at VR (Figure S5A). This alkaloid is rarely reported in *Lupinus* and appears that its occurrence is limited to North American species.^44,45^

Ammodendrine was present in *Lupinus* seeds at higher contents for D1, D2, and D3 (20.9–23.9 mg kg^–1^) than D4 (13.9 mg kg^–1^ DM, Figure S5B). In *L. albus* cv. Estoril D2 and D3 and *L. angustifolius* cv. Tango in D1 the content of ammodendrine was identical (3.81–4.37 mg kg^–1^ DM). The effect of location was verified only for *L. luteus* cv. Cardiga, the ammodendrine content in the seeds is higher at VR (23.3 mg kg^–1^ DM, Figure S5C). Ammodendrine, which derives from *L*-lysine, is known to co-occur in species containing quinolizidines and it has been assumed that it is an early byproduct of the biosynthesis of quinolizidines.^10,46^ The concentration for *L. albus* is below the values reported by de Cortes Sánchez et al.^47^ (110–270 mg kg^–1^) but of the same order of magnitude of the relative content observed by Święcicki et al.^37^ (1.04% of the total alkaloid content).

**Figure 6:**
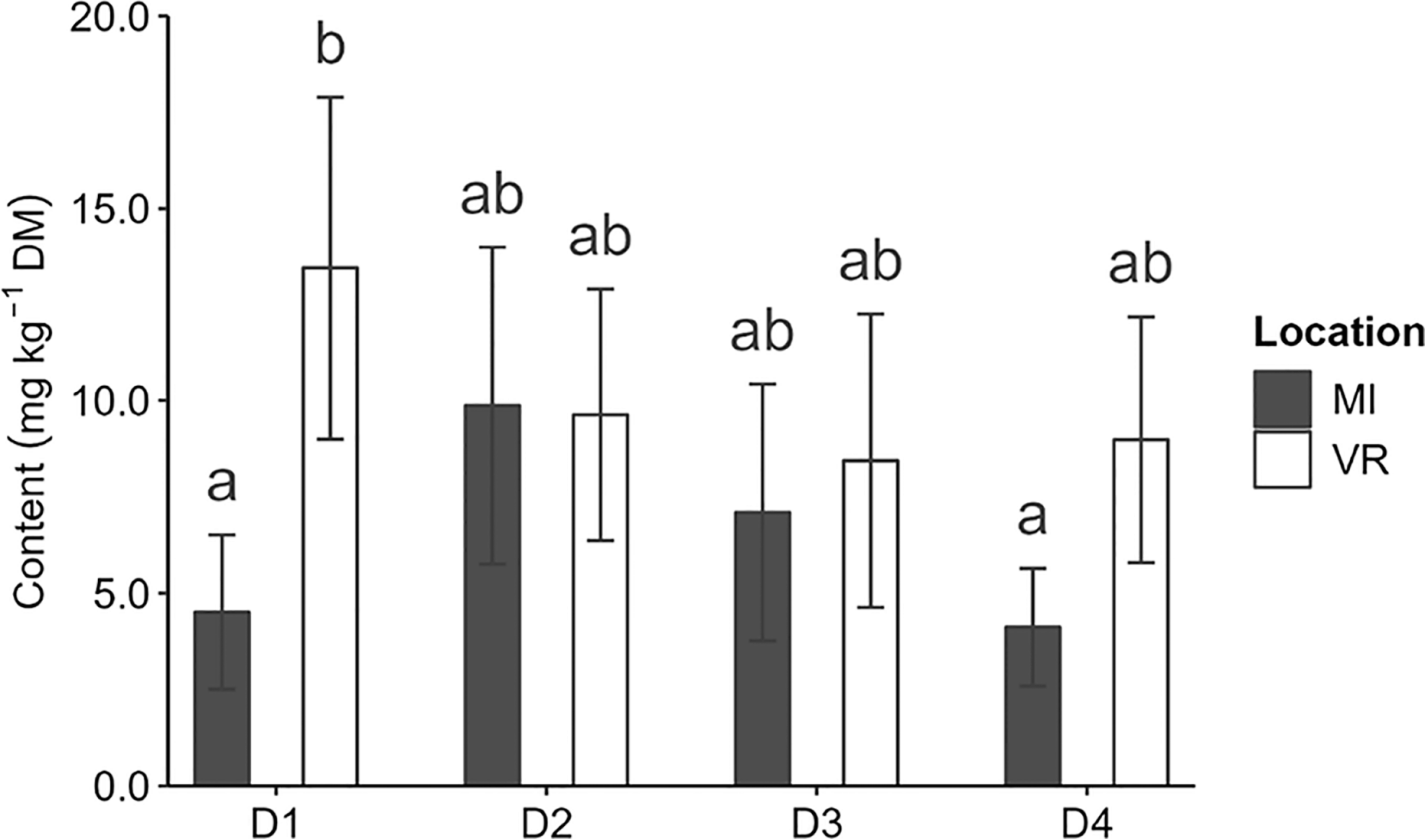
Effect of the interaction sowing date × location on piperidine alkaloids content (mg kg^–1^ DM) of *Lupinus* seed. Bars of the same factor not sharing the same letter differ significantly (*p* < 0.05).

As ammodendrine is the main piperidine alkaloid present in *Lupinus* seeds, the effects of sowing date and location on the total piperidine content were the same as those observed for ammodendrine. Moreover, a combined effect of sowing date × location interaction was observed for D1 (Figure 6); the content at MI (4.51 mg kg^–1^ DM) was lower than that at VR (13.4 mg kg^–1^ DM).

The safety concerns related to the occurrence of these alkaloids in food and feed are its teratogenicity and its high impact on the peripheral and central nervous systems.^43,48^ Also, it has been reported that *Lupinus* varieties with high *N*-methylammodendrine and ammodendrine are highly toxic for cattle.^45^

### Quinolizidine Alkaloids

Quinolizidine alkaloids were the group of alkaloids with the highest predominance in the studied *Lupinus* seeds at levels above 78% (w/w) of the total alkaloid content (Table S4). Quinolizidine alkaloids derive from the amino acid *L*-lysine, and at high levels are responsible for the bitter taste and toxicity to humans and animals.^6,10^

The total quinolizidine content varied significantly (*p* < 0.05, Table S7) with the genotype and location interaction. The lowest levels were obtained for the *L. albus* cv. Estoril (170–200 mg kg^–1^ DM) and *L. angustifolius* cv. Tango (38.5–204 mg kg^–1^ DM), regardless of location while the biosynthesis of quinolizidine alkaloids in *L. luteus* cv. Cardiga was higher for MI (2419 mg kg^–1^ DM) than for VR (1853 mg kg^–1^ DM, *p* < 0.05, Figure 4C). In Europe, no maximum levels of quinolizidine alkaloids content in food are defined^6^ nor in animal feed, even if it is defined that only sweet lupins can be used as animal feed, as specified in the Catalogue of feed materials.^49^ In Australia and New Zealand, the maximum limit for quinolizidine alkaloids in lupin products (lupin flour, lupin kernel flour, lupin kernel meal, and lupin hulls) for human consumption of 200 mg kg^–1^ is defined.^50^ Considering this value, the genotype *L. luteus* cv. Cardiga poses a high concern for human and animal consumption if used without any debittering process for alkaloid content reduction. The white (*L. albus* cv. Estoril) and narrow-leafed (*L. angustifolius* cv. Tango) genotypes showed considerably lower quinolizidine levels when compared to *L. luteus* genotype and close to that safety limit indicating the suitability for food or feed use with a previous debittering process.

In terms of the production of *Lupinus* for seeds’ consumption, it is important to consider the impact of edaphoclimatic characteristics in different locations on the alkaloids production in some *Lupinus* genotypes. Although species-specific responses to environmental conditions, it is reported that the quinolizidine alkaloid production is increased with the decrease of soil pH,^23^ potassium deficiency, and high levels of phosphorus.^24^ Considering the soil characteristics (Table S1) only the high soil phosphorus content at MI could be potentially one of the causes for the increased production of quinolizidines at this location; the pH and potassium content of soil at MI was higher than at VR. The climate factors such as rainfall and temperature can be also responsible for different abiotic stress responses of *Lupinus* plants.^3^ Although some species are stable to small variations in climate conditions, others can increase the biosynthesis of alkaloids due to water deficit and high temperatures.^3^ As MI showed lower rainfall and higher mean temperatures than VR (Figure S1).

Besides the differences in the total quinolizidine alkaloid content between the *Lupinus* genotypes, qualitative differences were observed as bicyclic quinolizidines were absent in *L. albus* and *L. angustifolius*, while tricyclic quinolizidines were absent in *L. luteus*; the three species showed tetracyclic quinolizidine alkaloids in different concentrations (Table S4).

Bicyclic quinolizidines were only significantly affected (*p* < 0.05, Table S4) by the genotype × sowing location and were only present in *L. luteus* cv. Cardiga, representing around 70% of the total alkaloid content in this genotype. The levels at MI (2206 mg kg^–1^ DM) higher than at VR (1664 mg kg^–1^ DM, Figure 4D), reflect the contents of lupinine (Figure S5D) and lusitanine (Figure S5E) that also showed higher levels at MI (2156 and 50.1 mg kg^–1^ DM, respectively). Being the main bicyclic quinolizidine and the most abundant alkaloid in *L. luteus* cv. Cardiga (c.a. 69.0% of the total alkaloid content) as verified by other authors for most of *L. luteus* varieties,^37,40,42^ lupinine content at MI (2156 mg kg^–1^ DM) was higher than at VR (1625 mg kg^–1^ DM). Lupinine showed inhibitory effects on nicotinic and muscarinic acetylcholine receptors, reduced the function of sodium and potassium channels, had uterine contracting effects, and influenced the conduction of stimuli in the heart, as reported by the German Federal Institute for Risk Assessment (BfR).^51^ Lusitanine determined in the range 38.9–50.1 mg kg^–1^ DM (1.7% of the total alkaloid content, Table S4), is rarely reported in the literature and, to the best of our knowledge, was first reported from trace levels (<1%) to 23% of the total alkaloids content in the *Lupinus* genus by Wink et al.,^8^ van Wyk et al.^52^

**Figure 7:**
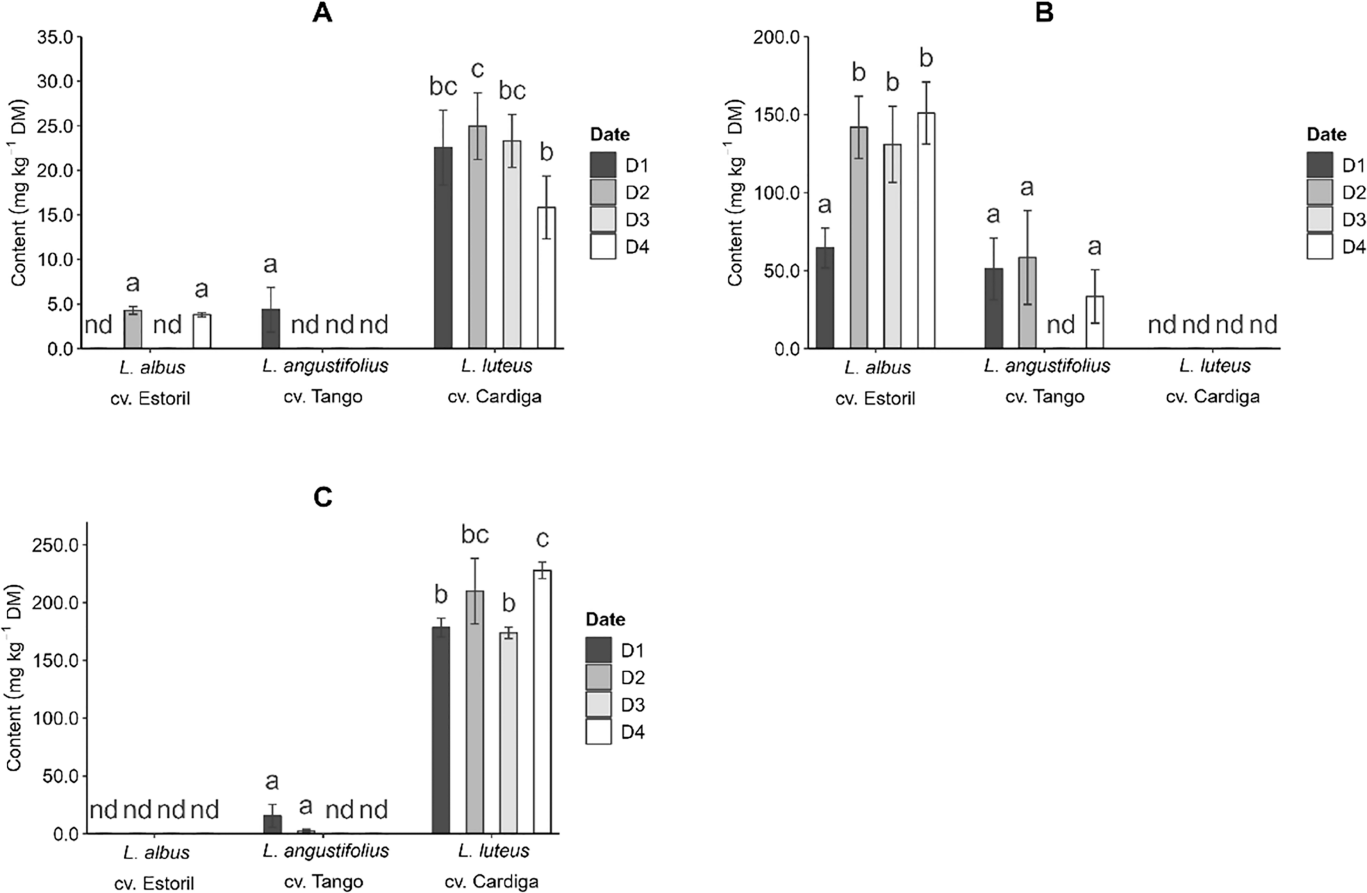
Effect of the interaction genotype × sowing date on (A) piperidine alkaloids, (B) tricyclic quinolizidine alkaloids, and (C) the sparteine content (mg kg^–1^ DM) of *Lupinus* seed. Bars of the same factor not sharing the same letter differ significantly (*p* < 0.05).

Tricyclic quinolizidine alkaloids are only present in *L. albus* cv. Estoril and *L. angustifolius* cv. Tango were affected (*p* < 0.05, Table S7) by the interactions species × sowing date and species × sowing location. The location affected only the *L. angustifolius* cv. Tango genotype as tricyclic quinolizidines was only present in plants sowed at MI (77.2 mg kg^–1^ DM, Figure 4E). For *L. albus* genotype, no effect (*p* > 0.05) of location was observed (116–128 mg kg^–1^ DM). The sowing date showed different impacts by genotype (Figure 7B). The content of tricyclic quinolizidine alkaloids in *L. angustifolius* cv. Tango was stable across dates D1, D2, and D4 (33.5–58.5 mg kg^–1^ DM) but was not detected at D3. For *L. albus* cv. Estoril the biosynthesis of these alkaloids was higher at latter sowing dates (D2 to D4, 131–151 mg kg^–1^ DM) than at D1 (64.5 mg kg^–1^ DM).

The individual alkaloids contributing to the class of tricyclic quinolizidine alkaloids are different between *L. albus* cv. Estoril and *L. angustifolius* cv. Tango, being the first characterized by the presence of albine and 11,12-seco-12,13-didehydromultiflorine (absent in *L. angustifolius* cv. Tango), and the last by the occurrence of angustifoline (Table 1).

In *L. albus* cv. Estoril, albine was the most abundant alkaloid in the seeds (86.8 mg kg^–1^, 45.9% of the total alkaloid content), and was not affected (*p* > 0.05, Table S7) either by sowing date nor location. Although lupanine has been reported as the main alkaloid in *L. albus* varieties,^8,40^ albine is one of the main alkaloids in *L. albus* and is present in variable concentrations (11.5–1767.4 mg kg^–1^) in different varieties.^40^ Yet, albine was also described in *L. angustifolius* and *L. luteus* but at trace levels.^40^

The other tricyclic quinolizidine was quantified in *L. albus* cv. Estoril, and 11,12-seco-12,13-didehydromultiflorine was affected (*p* < 0.001, Table S7) by the genotype × sowing date, being not detected for D1 and varying between 40.6 and 53.2 mg kg^–1^ DM for the remaining sowing dates (Figure S5F).

Angustifoline was detected only in *L. angustifolius* cv. Tango and showed significant (*p* < 0.05, Table S7) variations in its content with sowing date and location, being not detected at VR and at 67.0–117 mg kg^–1^ DM at MI for D1, D2, and D3 (Table S4). The drought stress, especially during the vegetative stage of the plant, was associated with increased angustifoline in *L. angustifolius* varieties.^20^ The lower rainfall at MI compared with VR (Figure S1) could promote the abiotic stress response mechanisms of the plants at that location. On the other hand, an interaction effect of water levels and temperature on the production of angustifoline can be present, and higher content of this alkaloid can be genotype-dependent.^53^ For this reason, the stability of genotypes across environments in terms of alkaloid production should be carefully evaluated.

Tetracyclic quinolizidine alkaloids were quantified in the three studied *Lupinus* species and showed to be affected (*p* < 0.05, Table S7) by the interaction genotype × sowing date × location.

The results (Figure 5I) showed that the sowing dates in different locations had an impact only on *L. angustifolius* cv. Tango. The content of tetracyclic quinolizidine alkaloids in *L. albus* cv. Estoril seeds varied between 17.8 and 132 mg kg^–1^ DM and between 170 and 249 mg kg^–1^ DM in *L. luteus* cv. Cardiga. A higher variation between the results was observed for *L. angustifolius* cv. Tango (4.08–247 mg kg^–1^ DM), with the highest values determined in seeds from MI D1 and MI D2 (202 and 247 mg kg^–1^ DM, respectively). These results show the high variability for some genotypes on the tetracyclic quinolizidine alkaloid content between environments as a result of distinct metabolic responses to abiotic stress conditions. Still, as each species is composed of *L*-lysine derived alkaloids resulting from different pathways,^54^ those variations should be discussed in terms of individual alkaloids. Generically, *L. albus* and *L. angustifolius* are characterized by lupanine-like alkaloids, while in *L. luteus* sparteine-like compounds occur (Table 1). Lupanine and sparteine have shown similar health effects to those verified for lupinine.^51^

In *L. luteus* cv. Cardiga, sparteine (198 mg kg^–1^ DM) and its isomer β-*iso*-sparteine (3.14 mg kg^–1^ DM) were determined in concentrations within the range observed for other European species.^40,42^ The occurrence of both lupinine and sparteine in *Lupinus* varieties can be explained by the formation pathways of these metabolites, as lupinine showed to be a side product of the synthesis of sparteine-like molecules from *L*-lysine.^10^ Sparteine was shown to be 2 to 3-fold more toxic to mice and pigs than lupanine.^6^ In humans, sparteine-sulfate can be used for cardiac arrhythmia treatment at the lowest oral dose equivalent to 0.16 mg kg^–1^ body weight.^6^ High doses of sparteine can have negative effects on the nervous system and a case of death for a dose of 30 mg kg^–1^ body weight was verified in a young child.^6^ Considering the high levels of sparteine-like alkaloids in *L. luteus* cv. Cardiga and their high toxicity; the seeds should be submitted to a debittering process before consumption. Sparteine was affected (*p* < 0.05, Table S7) by the interactions genotype × sowing date and genotype × location. The content of sparteine (2.53–15.5 mg kg^–1^ DM, Figure 7C) in *L. angustifolius* cv. Tango was not different (*p* > 0.05, Table S7) between dates D1 and D3; this alkaloid was not detected in samples from D3 and D4. In *L. luteus* cv. Cardiga seeds, the levels of sparteine (Figure 7C) were similar between dates D1, D2, and D3 (174–210 mg kg^–1^ DM) and between D2 and D4 (210 and 228 mg kg^–1^ DM). The sowing location had an effect only for the *L. angustifolius* genotype showing no detected sparteine at MI and 9.00 mg kg^–1^ DM at VR (Figure 4D). The yellow lupin genotype Cardiga showed no differences (*p* > 0.05) for sparteine content in seeds (186–209 mg kg^–1^ DM). For β-*iso*-sparteine, the interaction date × location was responsible for the variations observed (*p* < 0.05, Table S7) in *L. luteus* cv. Cardiga, the only genotype in which this alkaloid was present (Figure S5G). At MI, higher β-*iso*-sparteine contents were observed for D2 and D4 (4.46 and 4.36 mg kg^–1^ DM, respectively); at VR, similar contents were observed for D1, D2, and D4 (3.03–4.52 mg kg^–1^ DM) and no occurrence of the alkaloids was observed at D3.

The major tetracyclic quinolizidine alkaloids in *L. albus* cv. Estoril and *L. angustifolius* cv. Tango were lupanine (28.4 and 24.0 mg kg^–1^ DM, respectively) and 13α-hydroxylupanine (28.8 and 53.6 mg kg^–1^ DM, respectively, Table S4). Lupanine was affected (*p* < 0.005, Table S7) by the interaction genotype × location and only for *L. angustifolius* differences were observed (MI, 40.8 mg kg^–1^ DM and VR, 7.16 mg kg^–1^ DM, Figure 4E). The content of lupanine in *L. albus* cv. Estoril (27.7–29.0 mg kg^–1^ DM) was identical between locations and to *L. angustifolius* cv. Tango sowed at MI. For 13α-hydroxylupanine a significant (*p* < 0.05, Table S7) interaction of genotype × date × location was observed, and higher differences were registered for *L. angustifolius* cv. Tango (Figure S5H); both at MI and VR only for D1 and D2 13α-hydroxylupanine was quantified in the seeds, yet at MI the levels were considerably higher (145 and 194 mg kg^–1^ DM) when compared to VR (50.8 and 38.7 mg kg^–1^ DM), as was also observed for lupanine. The minor lupanine-derived alkaloids α-*iso*-lupanine and 13α-angelolyoxylupanine were also affected (*p* < 0.05, Table S7) by the triple interaction. The first alkaloid was detected in *L. albus* cv. Estoril (for D2, D3, and D4) and *L. angustifolius* cv. Tango seeds; the levels of α-*iso*-lupanine were identical for all of the sowing dates in *L. angustifolius* cv. Tango (5.88–9.53 mg kg^–1^ DM, Figure S5I). *Lupinus albus* cv. Estoril was the only genotype showing detectable levels of 13α-angelolyoxylupanine, with the highest content determined for MI D4 (6.25 mg kg^–1^ DM) compared to MI D2, MI D3 and VR D2 (3.59–4.19 mg kg^–1^ DM, Figure S5J). Multiflorine, with significant (*p* < 0.05, Table S7) variation with date, was quantified only in *L. albus* cv. Estoril for sowing date D2 (5.44 mg kg^–1^ DM, Table S4). This alkaloid has been described in *L. albus* cultivars seeds.^35,37,40,47,55^

### Productivity and Alkaloid Content Characterization

**Figure 8:**
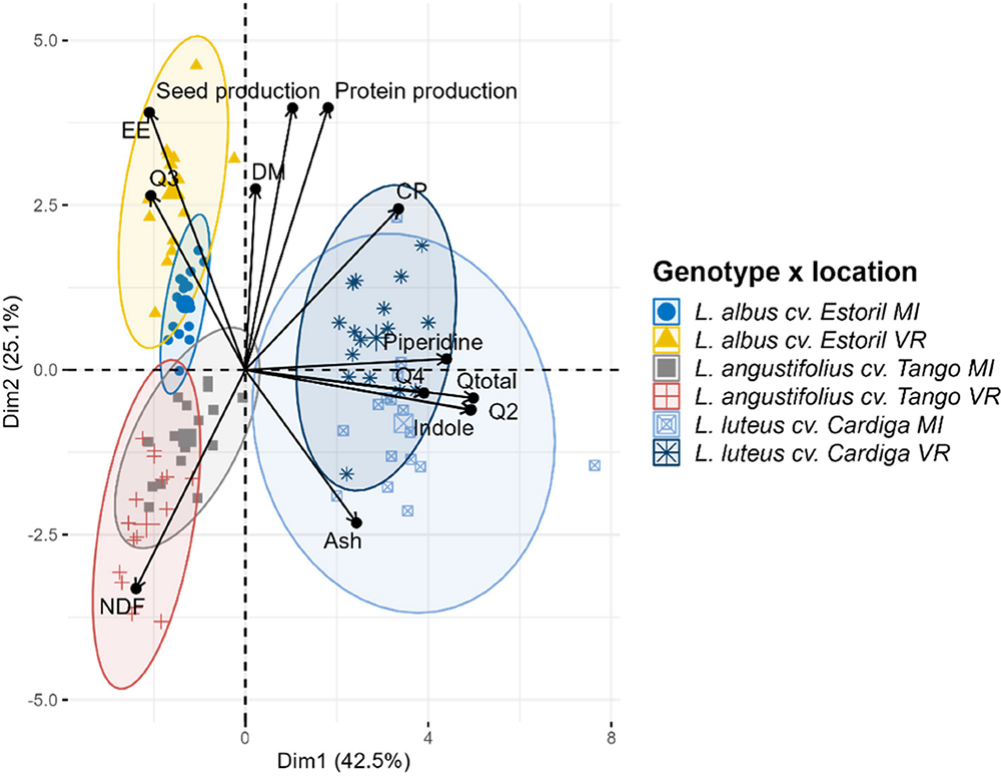
Principal Components Analysis (PCA) biplot of *Lupinus* seeds productivity (seed and protein), nutritional and antinutritional traits by genotype, and sowing location.

Generically the results of this trial showed a high contribution of the genotype, especially for the differences in the nutritional and antinutritional traits. Besides, the majority of the evaluated parameters showed a significant effect of the interaction genotype × sowing location. A PCA biplot showing the grouping of genotypes by location based on the productivity indexes (seed and protein), proximate chemical composition, and alkaloid content by class is presented in Figure 8.

The first two dimensions were responsible for 67.6% of the variation. The first dimension separated the species based on the qualitative and quantitative data of alkaloids. *Lupinus luteus* cv. Cardiga genotype is characterized by a higher content of indole, piperidine, bicyclic, tetracyclic, and total quinolizidine alkaloids; *L. albus* cv. Estoril distinguished by the content of tricyclic quinolizidine alkaloids. The second dimension of the PCA biplot allowed discrimination between locations and between *L. albus* cv. Estoril and *L. angustifolius* cv. Tango. The location VR was shown to be more suitable for increased seeds and protein production in *L. albus* cv. Estoril and *L. luteus* cv. Cardiga, showing the highest adaptability of these genotypes to the edaphoclimatic conditions of that location. Also, VR was demonstrated to increase the EE content of *L. albus* cv. Estoril, CP in *L. luteus* cv. Cardiga, and NDF in *L. angustifolius* cv. Tango. On the other hand, ash levels were increased in seeds sowed at MI in *L. luteus* cv. Cardiga.

In terms of genotype selection with both agronomic and food/feed interest, a combined analysis of the effects of edaphoclimatic conditions of productivity, nutritional, and antinutritional traits should be performed. In this context, *L. albus* cv. Estoril was the genotype with considerable seed and protein production while showing high CP content and having a quite reduced alkaloid content. In the conditions of the trial, a high effect of the sowing location was observed for productivities, nutritional traits, and alkaloid content at the expense of the sowing dates, which indicates a high effect of the climacteric conditions on the metabolic responses of the plants.

## Supporting Information Available

Soil characteristics, results of analysis of variance, climate data, and effects of genotype, sowing date, and sowing location on the studied traits (PDF)
