# Supplementary material for: Agronomic, Nutritional Traits, and Alkaloids of Lupinus albus, Lupinus angustifolius and Lupinus luteus Genotypes: Effect of Sowing Dates and Locations
Source: ACS Agric Sci Technol. 2024 Apr 2;4(4):450–62. doi: 10.1021/acsagscitech.3c00581 (PMC11022392; doi:10.1021/acsagscitech.3c00581)
Supplement: Supplementary file 1 — as3c00581_si_001.pdf [file as3c00581_si_001.pdf]

Supporting Information

Agronomic, nutritional traits, and alkaloids of *Lupinus albus*, *Lupinus angustifolius* and *Lupinus luteus* genotypes: effect of sowing dates and locations

Inês M. Valente[a,b *], André Monteiro[c], Carla Sousa[a], Carla Miranda[c,1], Margarida R. G. Maia[a], Carlos Castro[c], Ana R. J. Cabrita[a], Henrique Trindade[c], António J. M. Fonseca[a]

[a]REQUIMTE, LAQV, ICBAS, School of Medicine and Biomedical Sciences, University of Porto, Rua Jorge Viterbo Ferreira, 228, 4050-313 Porto, Portugal

[b]REQUIMTE, LAQV, Departament of Chemistry and Biochemistry, Faculty of Sciences, University of Porto, Rua do Campo Alegre 687, 4169-007 Porto, Portugal

[c]Centre for the Research and Technology of Agro-Environmental and Biological Sciences (CITAB), University of Trás-os-Montes and Alto Douro, Quinta de Prados, 5000-801 Vila Real, Portugal

[1]Present address: REQUIMTE, LAQV, University NOVA of Lisbon, Caparica, 2829-516 Lisbon, Portugal; Toxicology Research Unit (TOXRUN), University Institute of Health Sciences (IUCS), CESPU, CRL, 4585-116 Gandra, Portugal

*Corresponding Author:

Inês M. Valente

(e-mail) ines.valente@fc.up.pt

Table of Contents

Table S1. Soil characteristics in Mirandela (MI) and Vila Real (VR).

Table S2. Results of the analysis of variance (ANOVA) for seeds and protein production (t DM ha^{-1}) of *Lupinus* species seeds grown in 2 locations and 4 sowing dates.

Table S3. Results of the analysis of variance (ANOVA) for nutritional composition of *Lupinus* species seeds grown in 2 locations and 4 sowing dates.

Table S4. Individual alkaloids content (mg kg^{-1} DM) of *Lupinus* seeds by alkaloids' class, genotype, sowing location, and sowing date. Standard deviation is given between parentheses.

Table S5. Results of the analysis of variance (ANOVA) for indole alkaloids content of *Lupinus* species seeds grown in 2 locations and 4 sowing dates.

Table S6. Results of the analysis of variance (ANOVA) for piperidine alkaloids content of *Lupinus* species seeds grown in 2 locations and 4 sowing dates.

Table S7. Results of the analysis of variance (ANOVA) for quinolizidine alkaloids content of *Lupinus* species seeds grown in 2 locations and 4 sowing dates.

Table S8. Results of the analysis of variance (ANOVA) for the total alkaloids content of *Lupinus* species seeds grown in 2 locations and 4 sowing dates.

Figure S1. Monthly average minimum and maximum temperatures (˚C) and rainfall (mm) observed in Mirandela and Vila Real between August 2018 and July 2019. Long-term averages (LTA, between 1971 and 2000) are also presented.

Figure S2. Dry matter (DM, %) of *Lupinus* seeds. Effect of (A) genotype x location interactions and (B) sowing date. Bars of the same factor not sharing the same letter differ significantly ($p < 0.05$).

Figure S3. Effect of genotype x sowing date on the content of neutral detergent fibre (NDF, g 100 g^{-1} dry matter) of *Lupinus* seeds. Bars of the same factor not sharing the same letter differ significantly ($p < 0.05$).

Figure S4. Chemical structures of the alkaloids identified in the studied *Lupinus* species.

Figure S5. Effects on individual alkaloids content (mg kg^{-1} DM) of *Lupinus* seeds. Bars of the same factor not sharing the same letter differ significantly ($p < 0.05$). (A) *N*-methylammodendrine, (B and C) ammodendrine, (D) lupinine, (E) lusitanine, (F) 11,12-seco-12,13-didehydromultiflorine, (G) β-*iso*-sparteine, (H) 13α-hydroxylupanine, (I) α-*iso*-lupanine, and (J) 13α-angelolyoxylupanine.

Table S1. Soil characteristics in Mirandela (MI) and Vila Real (VR).*

	MI	***VR***
Particle size distribution (g kg^{-1})		
Coarse sand (200 - 2000 μm)	90 ± 13	210 ± 9
Fine sand (20 - 200 μm)	624 ± 10	387 ± 8
Silt (2 - 20 μm)	160 ± 7	275 ±10
Clay (<2 μm)	124 ± 13	126 ± 6
Chemical parameters		
pH (H_2O)	6.1 ± 0.1	4.8 ± 0.1
pH (KCl)	5.2 ± 0.3	3.9 ± 0.1
Organic matter (g kg^{-1})	11.0 ± 2.1	14.0 ± 0.7
Extractable P (mg P_2O_5 kg^{-1})	224.5 ± 28.3	67.0 ± 6.7
Extractable K (mg K_2O kg^{-1})	114.8 ± 16.7	85.5 ± 8.3
CEC (cmolc kg^{-1})		
Al	nd	0.68 ± 0.09
Ca	4.82 ± 0.42	2.49 ± 0.34
K	0.52 ± 0.51	0.26 ± 0.01
Mg	1.10 ± 0.08	0.68 ± 0.17
Na	0.13 ± 0.03	0.12 ± 0.01
Total CEC	6.31 ± 0.54	4.24 ± 0.29

*nd, not detected; CEC, Cation Exchange Capacity, cmolc kg^{-1}; centimol positive charge per kg of soil.

Table S2. Results of the analysis of variance (ANOVA) for seeds and protein production (t DM ha^{-1}) of *Lupinus* species seeds grown in 2 locations and 4 sowing dates.*

		Seed production				Protein production			
**Source of variation**	**df**	**SS**	**MS**	**p-value**	**%TRT**	**SS**	**MS**	**p-value**	**%TRT**
Genotype	2	16.78	8.39	**<0.001**	30.2	2.81	1.4044	**<0.001**	38.5
Date	3	9.782	3.261	**<0.001**	17.6	1.04	0.3456	**<0.001**	14.2
Location	1	2.844	2.844	**<0.001**	5.12	0.35	0.3482	**<0.001**	4.77
Genotype x Sowing date	6	2.619	0.437	0.063	4.71	0.41	0.0681	**0.021**	5.59
Genotype x Location	2	4.821	2.411	**<0.001**	8.68	0.58	0.2879	**<0.001**	7.89
Sowing date x Location	3	2.15	0.717	**0.021**	3.87	0.16	0.0537	0.105	2.21
Genotype x Sowing date x Location	6	1.609	0.268	0.272	2.90	0.138	0.023	0.494	1.89
Residuals	72	14.959	0.208			1.8234	0.0253		
Total	95	55.564	18.536			7.3005	2.5562		

*df, degree of freedom; SS, sum of squares; MS, mean squares; TRT, total sum of squares relative to main effects; statistically significant differences (p < 0.05) are marked in bold.

Table S3. Results of the analysis of variance (ANOVA) for nutritional composition of *Lupinus* species seeds grown in 2 locations and 4 sowing dates.*

		DM				Ash			
**Source of variation**	**df**	**SS**	**MS**	**p-value**	**%TRT**	**SS**	**MS**	**p-value**	**%TRT**
Genotype	2	6.13	3.0663	**0.004**	10.3	3.660	1.83	**<0.001**	18.3
Date	3	5.16	1.7193	**0.022**	8.64	0.876	0.29	**0.030**	4.39
Location	1	0.04	0.0417	0.774	0.07	3.992	3.99	**<0.001**	20.0
Genotype x Sowing date	6	2.49	0.4149	0.555	4.17	0.872	0.15	0.168	4.37
Genotype x Location	2	5.69	2.845	**0.005**	9.53	2.279	1.139	**<0.001**	11.4
Sowing date x Location	3	1.71	0.5705	0.341	2.86	0.343	0.114	0.302	1.72
Genotype x Sowing date x Location	6	2.24	0.3732	0.618	3.75	1.268	0.211	**0.045**	6.36
Residuals	72	36.24	0.5033			6.656	0.092		
Total	95	59.7	9.5342			19.946	7.815		

		EE				NDF			
**Source of variation**	**df**	**SS**	**MS**	**p-value**	**%TRT**	**SS**	**MS**	**p-value**	**%TRT**
Genotype	2	573.2	286.58	<0.001	85.6	2952.4	1476.200	**<0.001**	59.73
Date	3	1.3	0.45	0.194	0.19	28.5	9.500	0.601	0.58
Location	1	49.6	49.58	**<0.001**	7.41	162.2	162.200	**0.002**	3.28
Genotype x Sowing date	6	3.8	0.63	0.050	0.57	282.6	47.100	**0.009**	5.72
Genotype x Location	2	20.1	10.04	**<0.001**	3.00	280.6	140.3	**<0.001**	5.68
Sowing date x Location	3	0.9	0.29	0.379	0.13	49.8	16.6	0.358	1.01
Genotype x Sowing date x Location	6	0.6	0.09	0.912	0.09	92	15.3	0.427	1.86
Residuals	72	19.9	0.28			1094.8	15.2		
Total	95	669.4	347.94			4942.9	1882.4		

		CP			
**Source of variation**	**df**	**SS**	**MS**	**p-value**	**%TRT**
Genotype	2	2112.20	1056.1	**<0.001**	51.40
Date	3	9.80	3.3	0.864	0.24
Location	1	101.00	101	**0.007**	2.46
Genotype x Sowing date	6	162.80	27.1	0.071	3.96
Genotype x Location	2	606.5	303.3	**<0.001**	14.76
Sowing date x Location	3	65.9	22	0.185	1.60
Genotype x Sowing date x Location	6	94.2	15.7	0.326	2.29
Residuals	72	956.7	13.3		
Total	95	4109.1	1541.8		

*df, degree of freedom; SS, sum of squares; MS, mean squares; TRT, total sum of squares relative to main effects; DM, dry matter; EE, ether extract; NDF, neutral detergent fiber; CP, crude protein; statistically significant differences (p < 0.05) are marked in bold.

Table S4. Individual alkaloids content (mg kg^{-1} DM) of *Lupinus* seeds by alkaloids' class, genotype, sowing location, and sowing date. Standard deviation is given between parentheses. *

Location	Sowing date	Indole alkaloids	Piperidine alkaloids			Quinolizidine alkaloids																	Total alkaloids
						Bicyclic quinolizidine alkaloids			Tricyclic quinolizidine alkaloids				Tetracyclic quinolizidine alkaloids									
		Gramine /Total	Ammodendrine	*N*-methylammodendrine	Total	Lupinine	Lusitanine	Total	Albine	Angustifoline	11,12-seco-12,13didehydromultiflorine	Total	Sparteine	β-*iso*-sparteine	Multiflorine	α-*iso*-lupanine	Lupanine	alphahydroxylupanine	alphaAngelolyoxylupanine	Total	Total quinolizidines	
L. albus **cv. Estoril**																							
MI	***D1***	nd	nd	nd	nd	nd	nd	nd	55.9 (47.7)	nd	nd	55.9 (47.7)	nd	nd	nd	nd	22.1 (7.3)	18.7 (22.8)	nd	40.8 (28.5)	96.7 (74.7)	96.7 (74.7)
	D2	nd	3.71 (0.82)	nd	3.71 (0.82)	nd	nd	nd	90.3 (28.8)	nd	49.8 (12.3)	140 (40)	nd	nd	3.47 (0.70)	3.52 (0.28)	35.2 (1.4)	44.6 (14.9)	3.59 (0.31)	90.4 (15.7)	231 (56)	234 (56)
	D3	nd	nd	nd	nd	nd	nd	nd	93.0 (65.0)	nd	37.1 (24.8)	130 (89)	nd	nd	2.96 (2.17)	2.86 (1.97)	29.3 (10.6)	40.9 (28.6)	4.01 (2.80)	80.0 (45.7)	210 (133)	210 (133)
	D4	nd	3.93 (0.84)	nd	3.93 (0.84)	nd	nd	nd	137 (40)	nd	48.5 (4.6)	185 (39)	nd	nd	3.66 (0.29)	3.81 (0.40)	29.5 (2.3)	34.6 (9.6)	6.25 (1.15)	77.9 (10.1)	263 (37)	267 (38)
VR	***D1***	nd	nd	nd	nd	nd	nd	nd	73.0 (24.0)	nd	Nd	73.0 (24.0)	nd	nd	nd	nd	12.1 (2.0)	14.0 (16.6)	nd	26.1 (17.2)	99.2 (39.9)	99.2 (39.9)
	D2	nd	4.88 (1.46)	nd	4.88 (1.46)	nd	nd	nd	87.0 (45.6)	nd	56.7 (31.9)	144 (76)	nd	nd	7.40 (11.36)	nd	64.4 (87.4)	55.9 (46.9)	4.19 (1.72)	132 (147)	275 (222)	280 (224)
	D3	nd	nd	nd	nd	nd	nd	nd	87.7 (45.4)	nd	44.1 (10.8)	132 (56)	nd	nd	nd	nd	17.8 (7.8)	nd	nd	17.8 (7.8)	150 (64)	150 (64)
	D4	nd	3.68 (0.53)	nd	3.68 (0.53)	nd	nd	nd	71.0 (20.8)	nd	45.9 (31.9)	117 (52)	nd	nd	nd	nd	16.6 (3.9)	21.9 (15.4)	nd	38.4 (18.8)	155 (71)	159 (72)
L. angustifolius **cv. Tango**																							
MI	***D1***	nd	nd	nd	nd	nd	nd	nd	nd	102 (19)	nd	102 (19)	nd	nd	nd	6.56 (0.60)	50.2 (5.8)	145 (18)	nd	201 (21)	304 (39)	304 (39)
	D2	nd	nd	nd	nd	nd	nd	nd	nd	117 (88)	nd	117 (88)	nd	nd	nd	6.94 (2.87)	45.9 (23.6)	194 (108)	nd	247 (134)	364 (220)	364 (220)
	D3	nd	nd	nd	nd	nd	nd	nd	nd	nd	nd	nd	nd	nd	nd	5.88 (0.86)	31.0 (7.9)	nd	nd	36.9 (7.9)	36.9 (7.9)	36.9 (7.9)
	D4	nd	nd	nd	nd	nd	nd	nd	nd	67.0 (50.0)	nd	67.0 (50.0)	nd	nd	nd	9.53 (2.84)	36.0 (15.4)	nd	nd	45.6 (18.2)	113 (68)	113 (68)
VR	***D1***	nd	8.74 (8.06)	nd	8.74 (8.06)	nd	nd	nd	nd	nd	nd	nd	30.9 (34.8)	nd	nd	nd	8.33 (0.51)	50.8 (18.1)	nd	90.0 (41.5)	90.0 (41.5)	98.8 (49.0)
	D2	nd	nd	nd	nd	nd	nd	nd	nd	nd	nd	nd	5.06 (4.18)	nd	nd	nd	7.05 (1.97)	38.7 (12.3)	nd	50.8 (13.3)	50.8 (13.3)	50.8 (13.3)
	D3	nd	nd	nd	nd	nd	nd	nd	nd	nd	nd	nd	nd	nd	nd	nd	4.08 (1.11)	nd	nd	4.08 (1.11)	4.08 (1.11)	4.08 (1.11)
	D4	nd	nd	nd	nd	nd	nd	nd	nd	nd	nd	nd	nd	nd	nd	nd	9.19 (3.07)	nd	nd	9.19 (3.07)	9.19 (3.07)	9.19 (3.07)

***L. luteus* cv. Cardiga**																						
MI	***D1***	541 (69)	13.5 (3.8)	nd	13.5 (3.8)	1798 (263)	41.8 (16.4)	1840 (278)	nd	nd	nd	nd	174 (25)	2.43 (1.69)	nd	nd	nd	nd	nd	176 (27)	2016 (278)	2570 (341)
	D2	681 (213)	22.2 (14.4)	3.75 (1.04)	25.9 (14.9)	2279 (1019)	68.4 (41.6)	2347 (1055)	nd	nd	nd	nd	245 (108)	4.46 (1.83)	nd	nd	nd	nd	nd	249 (110)	2596 (1161)	3303 (1373)
	D3	522 (41)	21.3 (9.2)	nd	21.3 (9.2)	2085 (83)	46.8 (5.5)	2132 (83)	nd	nd	nd	nd	177 (10)	3.16 (0.71)	nd	nd	nd	nd	nd	181 (10)	2313 (79)	2856 (72)
	D4	593 (9)	8.41 (7.42)	nd	8.41 (7.42)	2463 (232)	43.3 (15.9)	2506 (234)	nd	nd	nd	nd	240 (24)	4.36 (1.43)	nd	nd	nd	nd	nd	244 (25)	2750 (257)	3351 (264)
VR	***D1***	439 (45)	28.3 (11.9)	3.33 (2.56)	31.6 (9.8)	1746 (139)	37.8 (8.1)	1784 (143)	nd	nd	nd	nd	183 (23)	3.17 (0.22)	nd	nd	nd	nd	nd	186 (23)	1970 (165)	2441 (205)
	D2	379 (21)	20.4 (5.7)	3.66 (0.44)	24.0 (6.1)	1484 (15)	32.6 (3.6)	1517 (17)	nd	nd	nd	nd	175 (9)	3.03 (0.12)	nd	nd	nd	nd	nd	178 (9)	1695 (22)	2098 (17)
	D3	370 (68)	25.3 (8.3)	nd	25.3 (8.3)	1514 (226)	38.0 (4.7)	1552 (230)	nd	nd	nd	nd	170 (18)	nd	nd	nd	nd	nd	nd	170 (18)	1722 (244)	2118 (315)
	D4	481 (10)	19.5 (6.4)	3.81 (1.47)	23.3 (5.5)	1755 (56)	47.1 (8.7)	1802 (64)	nd	nd	nd	nd	216 (3)	4.52 (0.36)	nd	nd	nd	nd	nd	220 (3)	2023 (64)	2527 (60)

*nd, not detected.

Table S5. Results of the analysis of variance (ANOVA) for indole alkaloids content of *Lupinus* species seeds grown in 2 locations and 4 sowing dates.*

		Gramine				Indole			
**Source of variation**	**df**	**SS**	**MS**	**p-value**	**%TRT**	**SS**	**MS**	**p-value**	**%TRT**
Genotype	2	5346721	2673360	**<0.001**	91.5	5346721	2673360	**<0.001**	91.5
Date	3	13936	4645	0.141	0.24	13936	4645	0.141	0.24
Location	1	74344	74344	**<0.001**	1.27	74344	74344	**<0.001**	1.27
Genotype x Date	6	27873	4645	0.096	0.48	27873	4645	0.096	0.48
Genotype x Location	2	148688	74344	**<0.001**	2.55	148688	74344	**<0.001**	2.55
Date x Location	3	17161	5720	0.083	0.29	17161	5720	0.083	0.29
Genotype x Date x Location	6	34322	5720	**0.043**	0.59	34322	5720	**0.042**	0.59
Residuals	72	178103	2474			178103	2474		
Total	95	5841148	2845252			5841148	2845252		

*df, degree of freedom; SS, sum of squares; MS, mean squares; TRT, total sum of squares relative to main effects; statistically significant differences (p < 0.05) are marked in bold.

Table S6. Results of the analysis of variance (ANOVA) for piperidine alkaloids content of *Lupinus* species seeds grown in 2 locations and 4 sowing dates.*

		***N*-methylammodendrine**				**Ammodendrine**				**Total**			
**Source of variation**	**df**	**SS**	**MS**	**p-value**	**%TRT**	**SS**	**MS**	**p-value**	**%TRT**	**SS**	**MS**	**p-value**	**%TRT**
Genotype	2	70.59	35.3	**<0.001**	34.1	7146	3573	**<0.001**	66.1	8636	4318	**<0.001**	68.6
Date	3	18.41	6.14	**<0.001**	8.90	105	35	0.326	0.97	142	47	0.180	1.13
Location	1	8.27	8.27	**<0.001**	4.00	237	237	0.006	2.19	333	333	**0.001**	2.64
Genotype x Sowing date	6	36.81	6.14	**<0.001**	17.8	541	90	0.011	5.01	491	82	**0.014**	3.90
Genotype x Location	2	16.54	8.27	**<0.001**	7.99	194	97	0.044	1.79	320	160	**0.005**	2.54
Sowing date x Location	3	8.8	2.93	**<0.001**	4.25	220	73	0.070	2.04	299	100	**0.019**	2.37
Genotype x Sowing date x Location	6	17.6	2.93	**<0.001**	8.51	222	37	0.294	2.05	337	56	0.078	2.68
Residuals	72	29.89	0.42			2143	30			2033	28		
Total	95	206.91	70.4			10808	4172			12591	5124		

*df, degree of freedom; SS, sum of squares; MS, mean squares; TRT, total sum of squares relative to main effects; statistically significant differences (p < 0.05) are marked in bold.

Table S7. Results of the analysis of variance (ANOVA) for quinolizidine alkaloids content of *Lupinus* species seeds grown in 2 locations and 4 sowing dates.*

Bicyclic quinolizidine alkaloids

		Lupinine				Lusitanine				Total			
	**df**	**SS**	**MS**	**p-value**	**%TRT**	**SS**	**MS**	**p-value**	**%TRT**	**SS**	**MS**	**p-value**	**%TRT**
Genotype	2	7.62×10^{7}	3.81×10^{7}	**<0.001**	91.4	42199	21100	**<0.001**	79.8	7.99×10^{7}	3.99×10^{7}	**<0.001**	91.3
Date	3	1.87×10^{5}	6.23×10^{4}	0.314	0.22	168	56	0.653	0.32	1.92×10^{5}	6.39×10^{4}	0.333	0.22
Location	1	7.53×10^{5}	7.53×10^{5}	**<0.001**	0.90	334	334	0.075	0.63	7.85×10^{5}	7.85×10^{5}	**<0.001**	0.90
Genotype x Sowing date	6	3.74×10^{5}	6.23×10^{4}	0.314	0.45	336	56	0.772	0.64	3.83×10^{5}	6.39×10^{4}	0.341	0.44
Genotype x Location	2	1.51×10^{6}	7.53×10^{5}	**<0.001**	1.80	668	334	**0.044**	1.26	1.57×10^{6}	7.85×10^{5}	**<0.001**	1.79
Sowing date x Location	3	2.21×10^{5}	7.38×10^{4}	0.242	0.27	594	198	0.132	1.12	2.32×10^{5}	7.72×10^{4}	0.251	0.26
Genotype x Sowing date x Location	6	4.43×10^{5}	7.38×10^{4}	0.216	0.53	1189	198	0.087	2.25	4.63×10^{5}	7.72×10^{4}	0.229	0.53
Residuals	72	3.72×10^{6}	5.17×10^{4}			7377	102			3.99×10^{6}	5.54×10^{4}		
Total	95	8.35×10^{7}	4.00×10^{7}			52865	22378			8.75×10^{7}	4.18×10^{7}		

Tricyclic quinolizidine alkaloids

		Albine				11,12-seco-12,13-didehydromultiflorine				Angustifoline				Total			
**Source of variation**	**df**	**SS**	**MS**	**p-value**	**%TRT**	**SS**	**MS**	**p-value**	**%TRT**	**SS**	**MS**	**p-value**	**%TRT**	**SS**	**MS**	**p-value**	**%TRT**
Genotype	2	160807	80403	**<0.001**	73.5	26510	13255	**<0.001**	53.6	27300	13650	**<0.001**	20.56	249242	124621	**<0.001**	52.1
Date	3	2146	715	0.310	0.98	4632	1544	**<0.001**	9.37	5431	1810	**0.010**	4.09	13612	4537	0.038	2.84
Location	1	547	547	0.338	0.25	21	21	0.683	0.04	13650	13650	**<0.001**	10.28	19037	19037	**0.001**	3.98
Genotype x Sowing date	6	4292	715	0.309	1.96	9264	1544	**<0.001**	18.7	10861	1810	**0.001**	8.18	39278	6546	**0.001**	8.20
Genotype x Location	2	1094	547	0.400	0.50	41	21	0.845	0.08	27300	13650	**<0.001**	20.56	23386	11693	**0.001**	4.88
Sowing date x Location	3	2559	853	0.236	1.17	47	16	0.943	0.10	5431	1810	**0.010**	4.09	7232	2411	0.204	1.51
Genotype x Sowing date x Location	6	5118	853	0.208	2.34	94	16	0.992	0.19	10861	1810	**0.001**	8.18	17973	2996	0.084	3.75
Residuals	72	42382	589			8817	122			31978	444			108978	1535		
Total	95	218945	85222			49426	16539			132812	48634			478738	173376		

Tetracyclic quinolizidine alkaloids

		Sparteine				β-*iso*-sparteine				α-*iso*-lupanine				Lupanine			
**Source of variation**	**df**	**SS**	**MS**	**p-value**	**%TRT**	**SS**	**MS**	**p-value**	**%TRT**	**SS**	**MS**	**p-value**	**%TRT**	**SS**	**MS**	**p-value**	**%TRT**
Genotype	2	813795	406898	**<0.001**	91.5	210.62	105.31	**<0.001**	70.3	215.1	107.55	**<0.001**	26.3	14926	7463	9.73E-08	25.32
Date	3	4368	1456	0.083	0.49	12.27	4.09	**<0.001**	4.10	16.42	5.47	**0.001**	2.01	2080	693	0.13847	3.53

Location	1	511	511	0.370	0.06	2.28	2.28	0.016	0.76	254.9	254.9	**<0.001**	31.2	3259	3259	0.0039	5.53
Genotype x Sowing date	6	12957	2159	**0.005**	1.46	24.53	4.09	**<0.001**	8.19	17.17	2.86	**0.008**	2.10	3640	607	0.14434	6.18
Genotype x Location	2	4311	2155	**0.038**	0.48	4.56	2.28	**0.004**	1.52	215.1	107.55	**<0.001**	26.3	5803	2901	0.00078	9.85
Sowing date x Location	3	3763	1254	0.123	0.42	6.12	2.04	**0.002**	2.04	16.42	5.47	**0.001**	2.01	641	214	0.62766	1.09
Genotype x Sowing date x Location	6	4466	744	0.325		12.24	2.04	**<0.001**		17.17	2.86	**0.008**		2219	370	0.42596	
Residuals	72	45298	629			26.92	0.37			64.48	0.9			26373	366		
Total	95	889469	415806			299.54	122.5			816.76	487.56			58941	15873		

		**Multiflorine**				**13α-hydroxylupanine**				**13α-angelolyoxylupanine**				**Total**			
**Source of variation**	**df**	**SS**	**MS**	**p-value**	**%TRT**	**SS**	**MS**	**p-value**	**%TRT**	**SS**	**MS**	**p-value**	**%TRT**	**SS**	**MS**	**p-value**	**%TRT**
Genotype	2	102.1	51.04	**<0.001**	14.4	46080	23040	**<0.001**	17.3	108.47	54.23	**<0.001**	33.4	348940	174470	**<0.001**	42.0
Date	3	42.6	14.2	0.063	6.02	39730	13243	**<0.001**	14.9	22.86	7.62	**<0.001**	7.04	73475	24492	**<0.001**	8.84
Location	1	1.2	1.21	0.643	0.17	14700	14700	**<0.001**	5.53	15.55	15.55	**<0.001**	4.79	49934	49934	**<0.001**	6.01
Genotype x Sowing date	6	85.2	14.2	**0.028**	12.0	59113	9852	**<0.001**	22.2	45.72	7.62	**<0.001**	14.1	93606	15601	**<0.001**	11.3
Genotype x Location	2	2.4	1.21	0.806	0.34	17661	8831	**<0.001**	6.64	31.1	15.55	**<0.001**	9.58	28628	14314	**0.005**	3.44
Sowing date x Location	3	23.9	7.96	0.243	3.38	6947	2316	**0.026**	2.61	21.44	7.15	**<0.001**	6.60	7101	2367	0.428	0.85
Genotype x Sowing date x Location	6	47.8	7.96	0.217		30937	5156	**<0.001**		42.88	7.15	**<0.001**		47650	7942	**0.009**	5.73
Residuals	72	402.7	5.59			50793	705			36.75	0.51			181991	2528		
Total	95	707.9	103.37			265961	77843			324.77	115.38			831325	291648		

		**Total quinolizidine alkaloids**			
**Source of variation**	**df**	**SS**	**MS**	**p-value**	**%TRT**
Genotype	2	83904926	41952463	**<0.001**	89.3
Date	3	391825	130608	0.153	0.42
Location	1	1549963	1549963	**<0.001**	1.65
Genotype x Sowing date	6	705064	117511	0.152	0.75
Genotype x Location	2	1242528	621264	**<0.001**	1.32
Sowing date x Location	3	305697	101899	0.247	0.33
Genotype x Sowing date x Location	6	633581	105597	0.204	0.67
Residuals	72	5202351	72255		
Total	95	93935935	44651560		

*df, degree of freedom; SS, sum of squares; MS, mean squares; TRT, total sum of squares relative to main effects; statistically significant differences ($p < 0.05$) are marked in bold.

Table S8. Results of the analysis of variance (ANOVA) for the total alkaloids content of *Lupinus* species seeds grown in 2 locations and 4 sowing dates.*

		Total alkaloids			
**Source of variation**	**df**	**SS**	**MS**	**p-value**	**%TRT**
Genotype	2	1.34×10^{8}	6.69×10^{7}	**<0.001**	90.4
Date	3	5.45×10^{5}	1.82×10^{5}	0.150	0.37
Location	1	2.25×10^{6}	2.25×10^{6}	**<0.001**	1.52
Genotype x Sowing date	6	8.52×10^{5}	1.42×10^{5}	0.210	0.58
Genotype x Location	2	2.17×10^{6}	1.09×10^{6}	**<0.001**	1.47
Sowing date x Location	3	4.48×10^{5}	1.49×10^{5}	0.220	0.30
Genotype x Sowing date x Location	6	8.61×10^{5}	1.44×10^{5}	0.210	0.58
Residuals	72	7.10×10^{6}	9.86×10^{4}		
Total	95	1.48×10^{8}	7.09×10^{7}		

*df, degree of freedom; SS, sum of squares; MS, mean squares; TRT, total sum of squares relative to main effects; statistically significant differences ($p < 0.05$) are marked in bold.

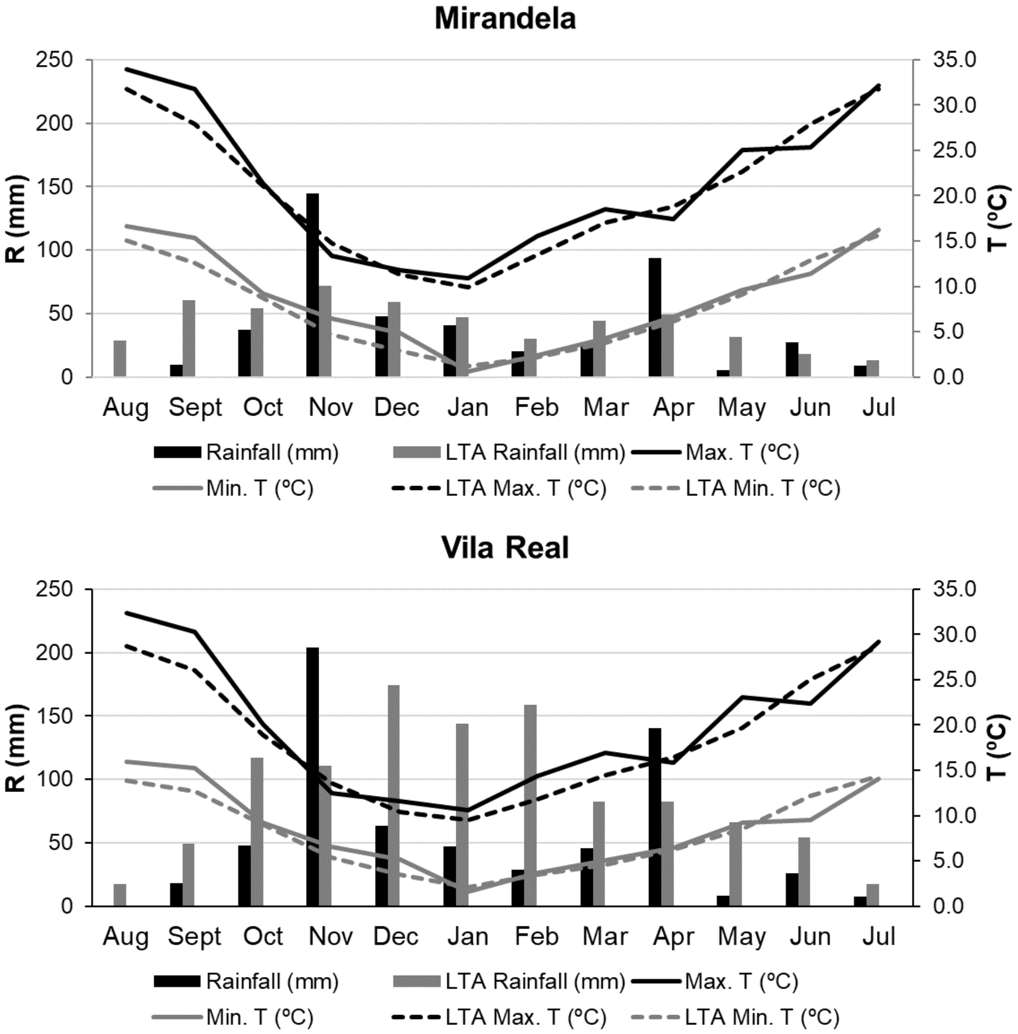

Figure S1. Monthly average minimum and maximum temperatures (˚C) and rainfall (mm) observed in Mirandela and Vila Real between August 2018 and July 2019. Long-term averages (LTA, between 1971 and 2000) are also presented.

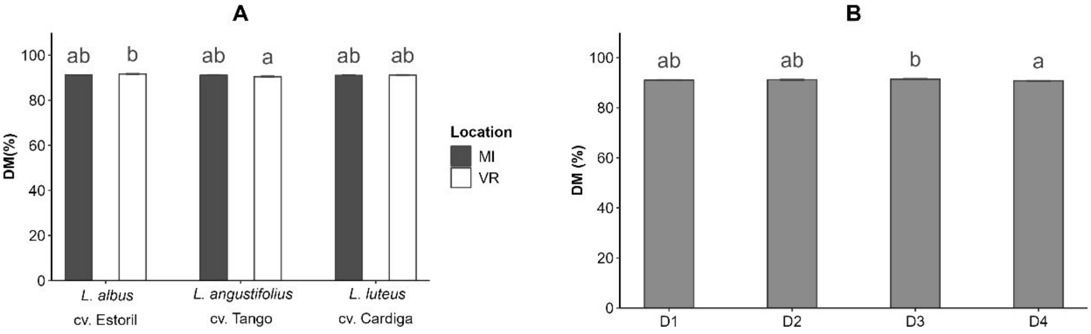

Figure S2. Dry matter (DM, %) of *Lupinus* seeds. Effect of (A) genotype x location interactions and (B) sowing date. Bars of the same factor not sharing the same letter differ significantly ($p < 0.05$).

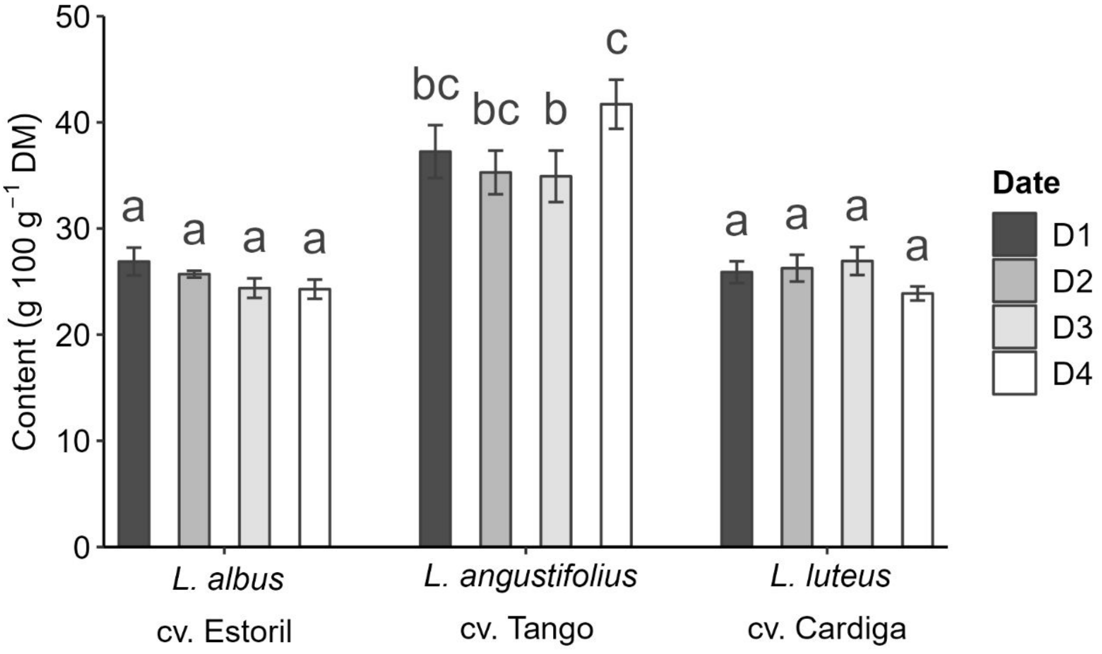

Figure S3. Effect of genotype x sowing date on the content of neutral detergent fibre (NDF, g 100 g^{-1} dry matter) of *Lupinus* seeds. Bars of the same factor not sharing the same letter differ significantly (p < 0.05).

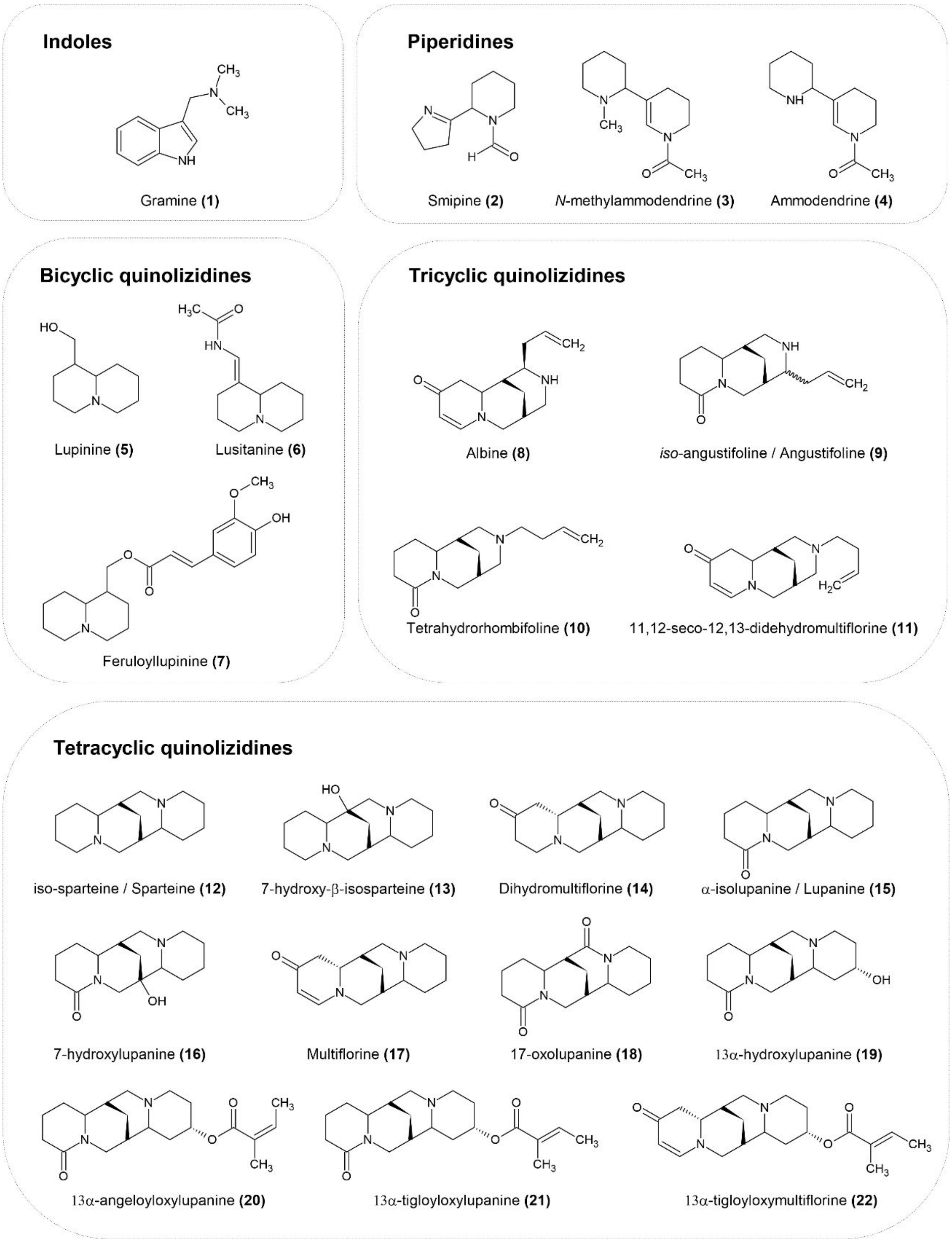

Figure S4. Chemical structures of the alkaloids identified in the studied *Lupinus* species.

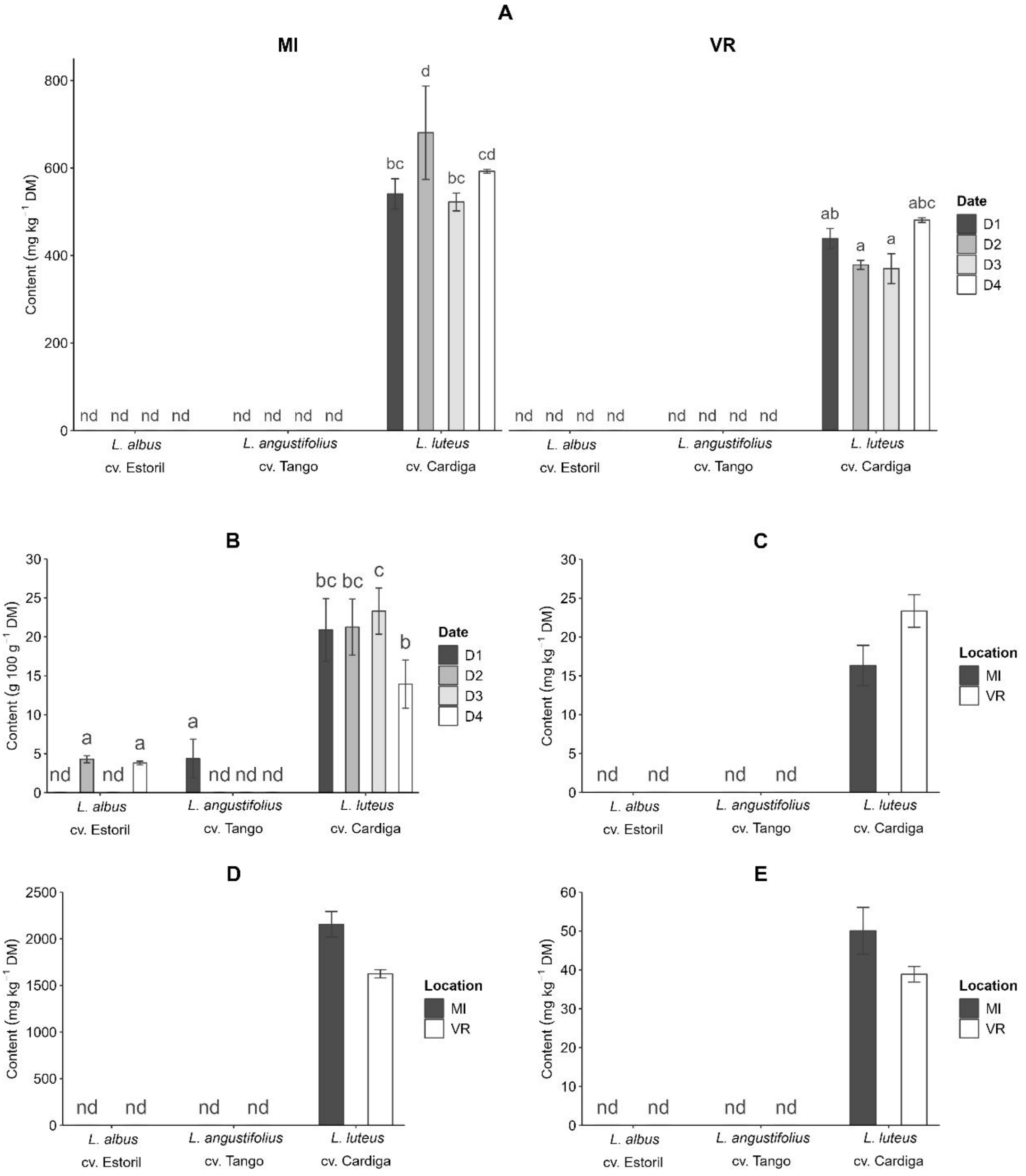
A
MI
VR
Content (mg kg -1 DM)
800
600
400
200
0
nd nd nd nd
nd nd nd nd
bc
d
bc
cd
ab
a
a
abc
Date
D1
D2
D3
D4
L. albus
cv. Estoril
L. angustifolius
cv. Tango
L. luteus
cv. Cardiga
B
Content (g 100 g -1 DM)
30
25
20
15
10
5
0
nd
a
nd
a
a
nd nd nd
bc bc
c
b
C
Content (mg kg -1 DM)
Location
MI
VR
nd nd nd nd
D
2500
2000
1500
1000
500
E
60
50
40
30
20
10

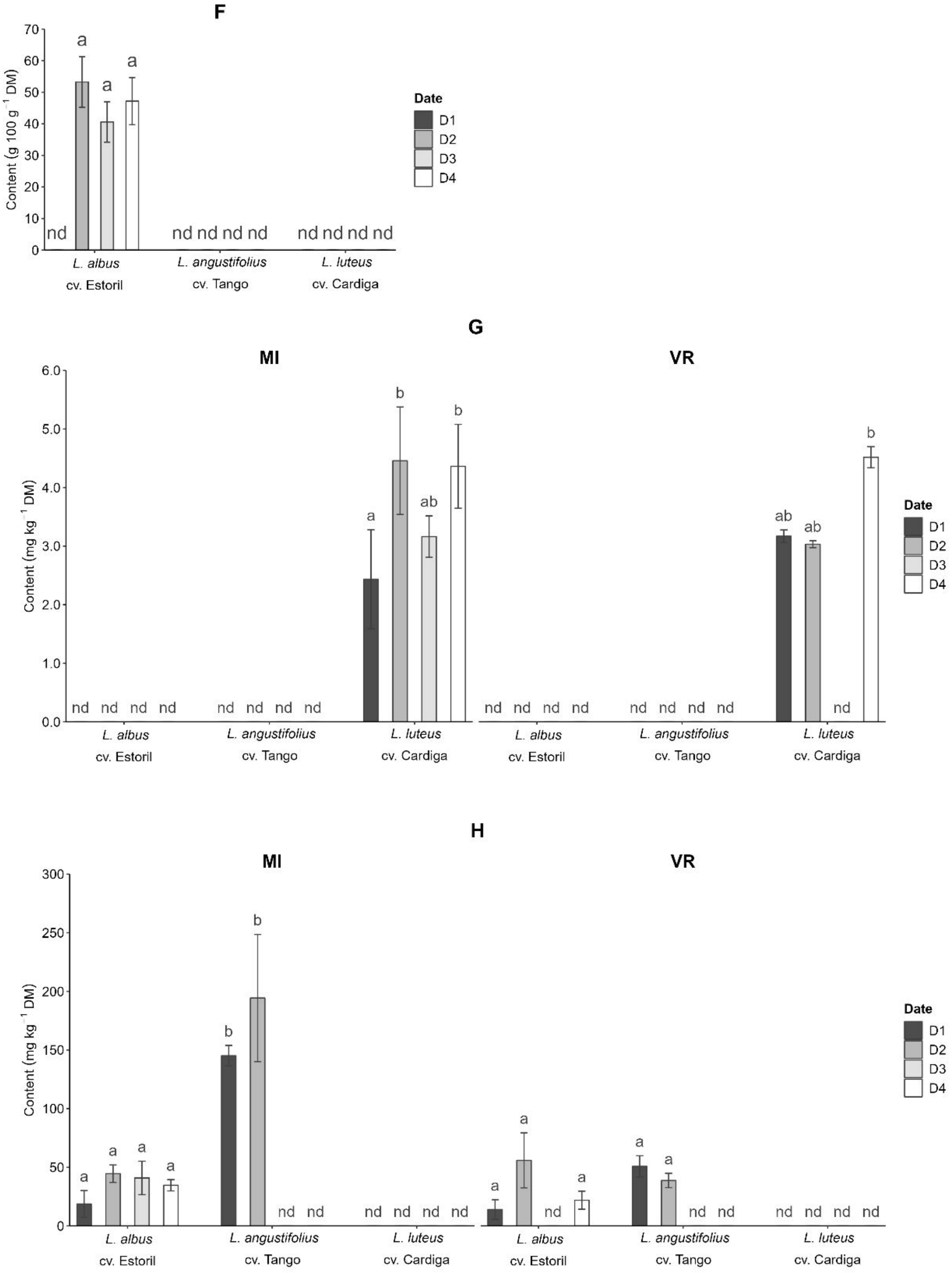
F
Content (g 100 g⁻¹ DM)
Date
D1
D2
D3
D4
nd
a
a
a
nd nd nd nd
nd nd nd nd
L. albus
cv. Estoril
L. angustifolius
cv. Tango
L. luteus
cv. Cardiga
G
MI
VR
Content (mg kg⁻¹ DM)
a
b
ab
b
ab
ab
nd
b
H
MI
VR
Content (mg kg⁻¹ DM)
a
a
a
a
b
b
nd
a
a
a

I

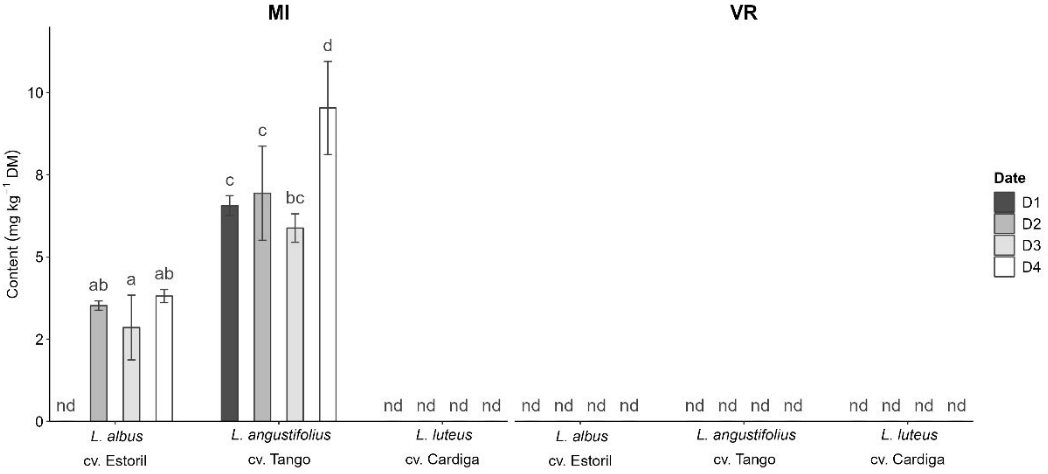

J

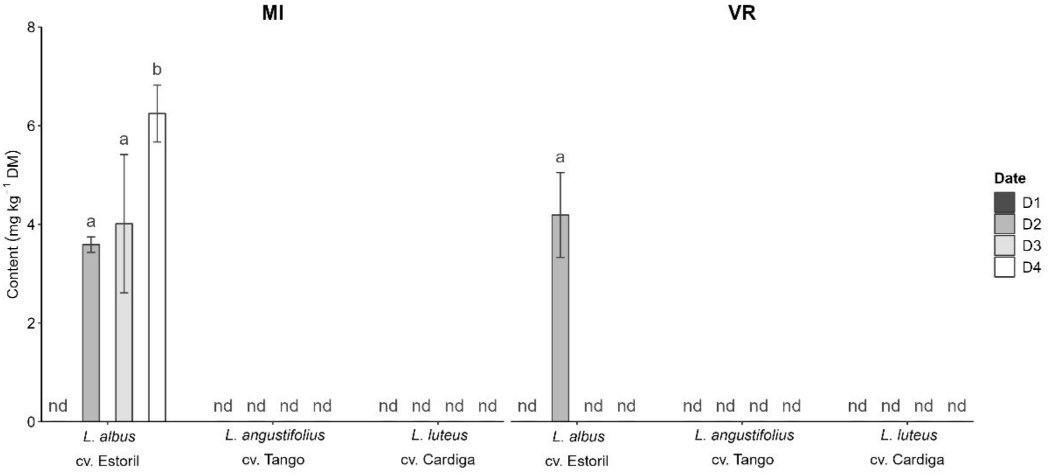

Figure S5. Effects on individual alkaloids content (mg kg^{-1} DM) of *Lupinus* seeds. Bars of the same factor not sharing the same letter differ significantly ($p < 0.05$). (A) *N*-methylammodendrine, (B and C) ammodendrine, (D) lupinine, (E) lusitanine, (F) 11,12-seco-12,13-didehydromultiflorine, (G) β-*iso*-sparteine, (H) 13α-hydroxylupanine, (**I**) α-*iso*-lupanine, and (J) 13α-angelolyoxylupanine.
